# Pooches on a platform: Text mining twitter for sector perceptions of dogs during a global pandemic

**DOI:** 10.3389/fvets.2023.1074542

**Published:** 2023-03-01

**Authors:** Kirsten M. McMillan, Katharine L. Anderson, Robert M. Christley

**Affiliations:** Dogs Trust, London, United Kingdom

**Keywords:** dog, companion animal, COVID-19, Twitter, web scraped data, text mining, sentiment analysis

## Abstract

**Introduction:**

Businesses commonly text mine Twitter data to identify patterns and extract valuable information. However, this method is rarely applied to the animal welfare sector. Here, we describe Twitter conversations regarding dogs during a global pandemic, assess the evolution of sentiment, and examine the dynamics of sector influence.

**Methods:**

Between March and August 2020, we gathered 61,088 unique tweets from the United Kingdom and Republic of Ireland, relating to COVID-19 and dogs. Tweets were assigned to one of four pandemic phases and active accounts were assigned to a sector: Personal (i.e., UK and ROI public), Press (i.e., mass media), State (i.e., Government, Police, and NHS), and Other (i.e., welfare organizations, social enterprises, research organizations, charity, and business).

**Results:**

Word frequency and sentiment analysis between phases and sectors were assessed, and cross correlation functions and lagged regressions were used to evaluate sector influence. Topical foci of conversations included: meat trade, separation anxiety and dog theft. Sentiment score remained stable until the last phase where sentiment decreased (*F*_3, 78, 508_ = 44.4, *p* < 0.001), representing an increased use of negative language. Sentiment differed between the four sectors (*F*_3, 11, 794_ = 52.2, *p* < 0.001), with Personal and Press accounts presenting the greatest use of negative language. Personal accounts were initially partly influenced by State accounts (*R* = −0.26; *p* = 0.05), however this altered to Press accounts by the last phase (*R* = −0.31; *p* = 0.02).

**Discussion:**

Our findings highlight that whilst Personal accounts may affect sector-specific messaging online, perhaps more importantly: language used, and sentiment expressed by Press, State and Other accounts may influence public perception. This draws attention to the importance of sector responsibility regarding accurate and appropriate messaging, as irresponsible/ill-considered comments or campaigns may impact future human-animal interaction.

## Introduction

With the rapid development of the internet and mobile networks, social media platforms have grown quickly (1), creating a vast source of raw data. Computational tools, such as text mining, allow researchers to collate and analyze user-generated language data, providing insight into online behavior and cohort opinion. Whilst charities may use these platforms to help promote their message and gain supporters, they rarely take advantage of computational tools to better understand their audience. As one of the most powerful social media platforms, Twitter allows both consumers and brands to deliver insights and optimize engagement (2). Here, we argue that these tools may provide benefit to the animal welfare sector, as social media creates an environment where animals are represented within human society.

Twitter, established in 2006, is a microblogging social media platform that allows users to “tweet” content publicly, in 280 characters or less. With ~330 million active users, ranging from personal to government sanctioned accounts, Twitter is arguably the most utilized short-text discussion forum in the world (1). It can disseminate and reflect information broadly and rapidly, allowing for single or multi-way communication. Text mining (3), i.e., the ability to search for text or hashtags (keywords related to a topic that are proceeded by #), enables researchers to examine Twitter archives and extract relevant information. This not only allows for informed decision-making by highlighting current areas of interest or concern, but it also provides situational awareness and flexibility, which may be of particular interest at present due to the unprecedented impacts of the novel coronavirus termed COVID-19 [caused by SARS-CoV-2 (4)].

Sentiment analysis is a text mining technique that uses machine learning and natural language processing to examine the content of free text for the intensity of positive and negative opinions and emotions. Computerized packages have been developed that automate the process, allowing large numbers of free-text comments to be quickly processed into quantitative sentiment scores. Lexicon-based sentiment analysis methods, e.g., *nrc, afinn*, and *bing*, have become very popular due to their unsupervised nature and easy-use properties (5). The immediate nature of Twitter feeds enables communication in real-time between peers; government and public sector; companies and suppliers; industry and consumers; charities and supporters; and many more, providing a wide-ranging source of user-generated language data. While businesses may use Twitter as a cost-effective method of engaging with their consumer base, personal accounts may present more emotive language, possibly supplemented by information sourced from press or government accounts. Consequently, language used, and sentiment presented may vary across sectors, which in turn, may have a critical impact on the behavior of others, particularly during times of crisis (6). Understanding the influence that sectors have on public perception, and *vice versa*, is imperative to animal welfare issues and those working within the field, as this will impact upon public engagement and future policy making. Whilst text mining and sentiment analysis of social media data has been widely employed within multiple fields, such as politics (7, 8), disaster monitoring and response (9, 10), ecological modeling (11, 12), and human health (13, 14), this tool remains relatively underutilized within animal welfare (15, 16).

As a result of the COVID-19 pandemic, countries world-wide have faced difficulties ranging from human health services (17) to animal conservation (18). Three main impacts have been suggested regarding animal welfare (19): (1) an immediate impact due to sudden human confinement and inactivity; (2) a medium to long-term impact due to the effects of the resulting economic crisis on farming and veterinary services; and (3) an increased attention to the public health implications of coronavirus infections in animals, including those found wild (20), in farms (21), and those living with humans, i.e., pets (22). With regards to pet dogs, there is growing concern regarding the impact that the pandemic has had on multiple aspects of their welfare (23–27). These include (but are not limited to): deviations from the daily routine, increasing the probability of a pet dog developing behavioral issues; veterinary visits falling due to real or perceived difficulty in accessing veterinary care; and an increased demand for puppies and associated intensification of large-scale breeding and/or rise of illegal puppy smuggling. Tracking main topics discussed online and assessing sentiment surrounding these topics may be beneficial to multiple stakeholders as it allows for the development of proactive response strategies (28).

To ensure progress within the field, it is imperative that methods of assessing and measuring topics of concern are continually identified and developed. As far as the authors are aware, no study has attempted to examine sentiment expressed by specific sectors on social media, within the context of animal welfare. Given the low-cost of data utilization, ability to search archives for relevant topics and real-time streaming nature of Twitter, these methods may provide a unique, yet untapped, tool for the animal welfare sector. Consequently, we focused on canine welfare as an example topic, to assess the functionality of applying text mining and sentiment analyses to tweets, in order to gain meaningful temporal and sector specific insight. More specifically, we aimed to: (1) highlight the topical foci of conversations regarding canine-COVID topics in the United Kingdom and Republic of Ireland, (2) assess temporal evolution of sentiment (throughout key phases of the pandemic), and (3) assess and compare sentiment expressed by specific sectors (“Personal”, “Press”, “State”, and “Other”) and examine their influence on each other.

## Materials and methods

### Data collection

Implementing R package *rtweet* (29), we connected with the official Twitter application programming interface (API) and gathered English language tweets from the United Kingdom (UK) and the Republic of Ireland (ROI) by applying a geocode buffer (Lat = 55.5166, Long = −4.0661, Buffer = 390 miles; Supplementary Figure 1). Tweets were searched using two term lists: (1) words related to COVID-19 and (2) words related to dogs. Tweets where ≥1 search term(s) from both lists were present, were included in the analysis. All search terms and their reported frequencies are listed in Supplementary Table 1. The search engine relies on Twitter's search API and is limited to tweets published in the past 7 days. Consequently, the script was run weekly. We began running the script on the March 27th, 2020, purging retweets and storing backup datafiles incrementally, in order to preserve historical data (further details on dates included below).

### Data cleaning and manipulation

Data analysis was performed with the statistical software R (30) (version 4.0.2), using the aforementioned package *rtweet* (29), as well as *dplyr* (31)*, stringr* (32), and *tidyverse* (33) for data cleaning and manipulation. Packages *tidytext* (34) and *tm* (35) were used for text mining. For the production of figures, *ggplot2* (36) was implemented, along with *wordcloud* (37).

A profane word list was imported (38) and augmented by the authors, to include 750 English terms that could be found offensive. Profanities were then removed from tweets, as sentiment of profane language can be positive or negative, depending on context. For example, profanity may co-occur with insults or abusive speech, or may be used in tweets to express emphasis regarding positive sentiment. Duplicate tweets were identified and removed, by matching Twitter handle (i.e., account name), content of tweet and date. To ensure that the tweet was relevant, i.e., referred to the COVID-19 and dogs, relevance was checked by searching for the presence of one or more pre-defined terms (Supplementary Table 2). Tweets that did not include any relevant words were excluded from the analysis.

**Figure 1:**
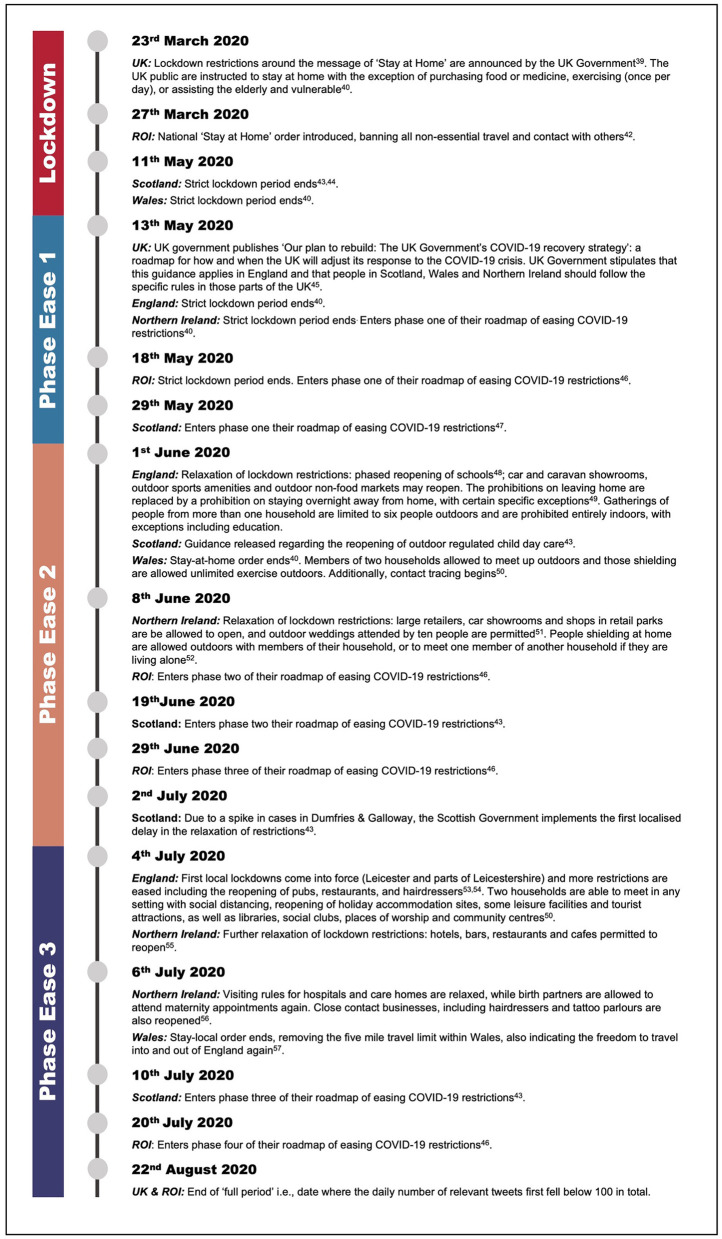
Dates of major events occurring across the UK nations (England, Northern Ireland, Wales, and Scotland) and Republic of Ireland (ROI), during the full period (March 27th–August 22nd, 2020), which are relevant to determining the four pandemic phases [Lockdown, Phase Ease 1, Phase Ease 2, and Phase Ease (39, 40, 42–57)].

The UK entered lockdown on March 23rd, 2020 (39). Data collection for this research commenced on the March 27th, 2020, succeeding reactive project initiation and planning. Data collection ended on the August 22nd, 2020: the date where the daily number of relevant tweets first fell below 100 in total. This period will now be referred to as the “full period”. Within the full period, tweets were assigned to one of four phases: (1) Lockdown (LD; March 27th–May 12th, 2020), (2) Phase Ease 1 (PE1; May 13th–May 31st, 2020), (3) Phase Ease 2 (PE2; June 1st–July 3rd, 2020), and (4) Phase Ease 3 (PE3; July 4th–August 22nd, 2020). Following LD, the devolved nations (Northern Ireland, Wales, and Scotland) and ROI followed their own paths with regards to determining restrictions and, in turn, easing restrictions: based on their respective number of cases and regional NHS capacity (40). Unfortunately, whilst data were reliably collected from the UK and ROI, tweet location could not be assigned to country as most Twitter users opt to keep their location unpublished (41). Consequently, for the purposes of this study, we broadly determined four pandemic phases based on major events occurring across the UK and ROI (39, 40, 42–57), details for which are outlined in Figure 1.

Twitter accounts where 4 or more relevant tweets were posted during the full period, were assigned to one of four sectors: (1) ‘Personal', i.e., personal accounts/UK and ROI public; (2) “Press”, i.e., all variations of mass media; (3) “State”, i.e., Government, Police, and NHS; and (4) “Other”, i.e., all other sectors such as (but not limited to) animal welfare organizations, social enterprises, research organizations, charity and business. Sector categorization was carried out *via* manual assessment of the Twitter account “Bio”: a small public summary regarding account holder or business. The probability of these accounts functioning as bots or bot accounts, i.e., automated programs, were assessed using R package *tweetbotornot* (58). Legitimate bots may generate a large number of benign tweets delivering news and updating feeds, while malicious bots may spread spam, incorrect and/or irresponsible content (59). However, this was for reference only, and no data were excluded due to the outcome. Limitations of the above methodologies are discussed in Supplementary Note 1.

### Word frequency and negation

Word frequency was assessed and compared using the full corpus of tweets (tweet content and hashtag/s), where punctuation, stop words (i.e., commonly used words which do not add much meaning to a sentence, e.g., “the”, “an”, “in”, “just”, “can” etc.) and numbers were removed. Words were then stemmed, i.e., reducing inflected or sometimes derived words to their word stem, base, or root form (e.g., the words *fishing, fished*, and *fisher* to the stem *fish*). Additionally, due to sampling strategy, all words used in the initial API Twitter search (those listed in Supplementary Table 1), were also removed from the corpus, as their high frequency was inevitable.

Tokenizing at the word level can help greater understanding of the most frequently used words. However, examining different units of text (e.g., consecutive words) may provide a greater understanding of topics discussed. As such, we considered both single words (or tokens) and bigrams (two consecutive words) for both sector and/or phase. Additionally, bigrams provide further context in sentiment analysis, as it allows for the quantification of words preceded by negation. Negating words incorporated in analyses include: “aren't”, “can't”, “didn't”, “don't”, “hadn't”, “hasn't”, “no”, “not”, “shouldn't”, “wasn't”, “without”, and “won't”. This highlights tokens which should be considered with caution. Pearson correlation coefficient was applied to test correlation of word use between phases and sectors.

### Weighted log odds ratios

Log odds ratios were used to compare word usage between sectors and/or phases. The outcome, two “lexical histograms”, are taken from two sources X and Y, whose patterns of usage are contrasted. This method takes into account the likely sampling error in counts, discounting differences that are probably accidental, and enhances differences that are genuinely unexpected given the null hypothesis that both X and Y are making random selections from the same vocabulary. These features enabled differences in very frequent words to be detected.

### Term frequency—Inverse document frequency

TF-IDF is a numerical statistic that evaluates how relevant a word is to a document in a collection of documents. This is done by multiplying two metrics: how many times a word appears in a document, and the inverse document frequency of the word across a set of documents. The logic of TF-IDF is that the words containing the greatest information about a particular document are the words that appear many times in that document, but in relatively few others. This was used to identify distinctive word use within a given sector and/or phase.

### Sentiment analysis

Sentiment analysis research has been accelerated with the development of several lexical resources. A wide variety of methods and dictionaries exist for evaluating the opinion or emotion in text. However, the aforementioned *tidytext* package provides access to several sentiment lexicons, including three general-purpose lexicons: *afinn* (60), *nrc* [syuzhet (61)], and *bing* (62). All three lexicons contain many English words and are based on tokens, i.e., single words. These words are assigned scores for positive/negative sentiment. The *afinn* lexicon assigns words with a score of −5 to 5, with negative scores indicating negative sentiment and positive scores indicating positive sentiment. The *nrc* lexicon categorizes words into the following: positive, negative, anger, anticipation, disgust, fear, joy, sadness, surprise, and trust. The *bing* lexicon categorizes words in a binary fashion, into positive and negative categories. Due to the variation in scoring, all three lexicons were implemented in this project, to maximize opportunity to explore variation in measures of sentiment temporally and between sectors. Variations in sentiment were compared between sector and/or phase using univariate ANOVA. Any significant results from ANOVA testing were further tested using Tukey *post-hoc* test to determine which categorical groupings were different from the others.

### Cross correlation functions and lagged regressions

In order to assess the relationship between two time series (*y*_*t*_*andx*_t_), the series y_t_ may be related to past lags of the *x*-series. To do so, the cross-correlation function [using function CCF, *tseries* package (63)] was implemented to determine the correlation between pair wise time series, as a function of the time lag or delay (64). This produces correlation coefficients between two time-series data at each lag. Consequently, we identified sectors which may influence others by assessing lags of mean sentiment within one sector that may be useful predictors of another. Relations between two sectors were then analyzed by means of Pearson correlations [using the function cor.test, *stats* package (30)], including the lagged period and correlations displayed using *DiagrammeR* package (65).

### Ethics

Ethical approval for this study was granted by Dogs Trust Ethical Review Board (Reference Number: ERB036).

## Results

**Figure 2:**
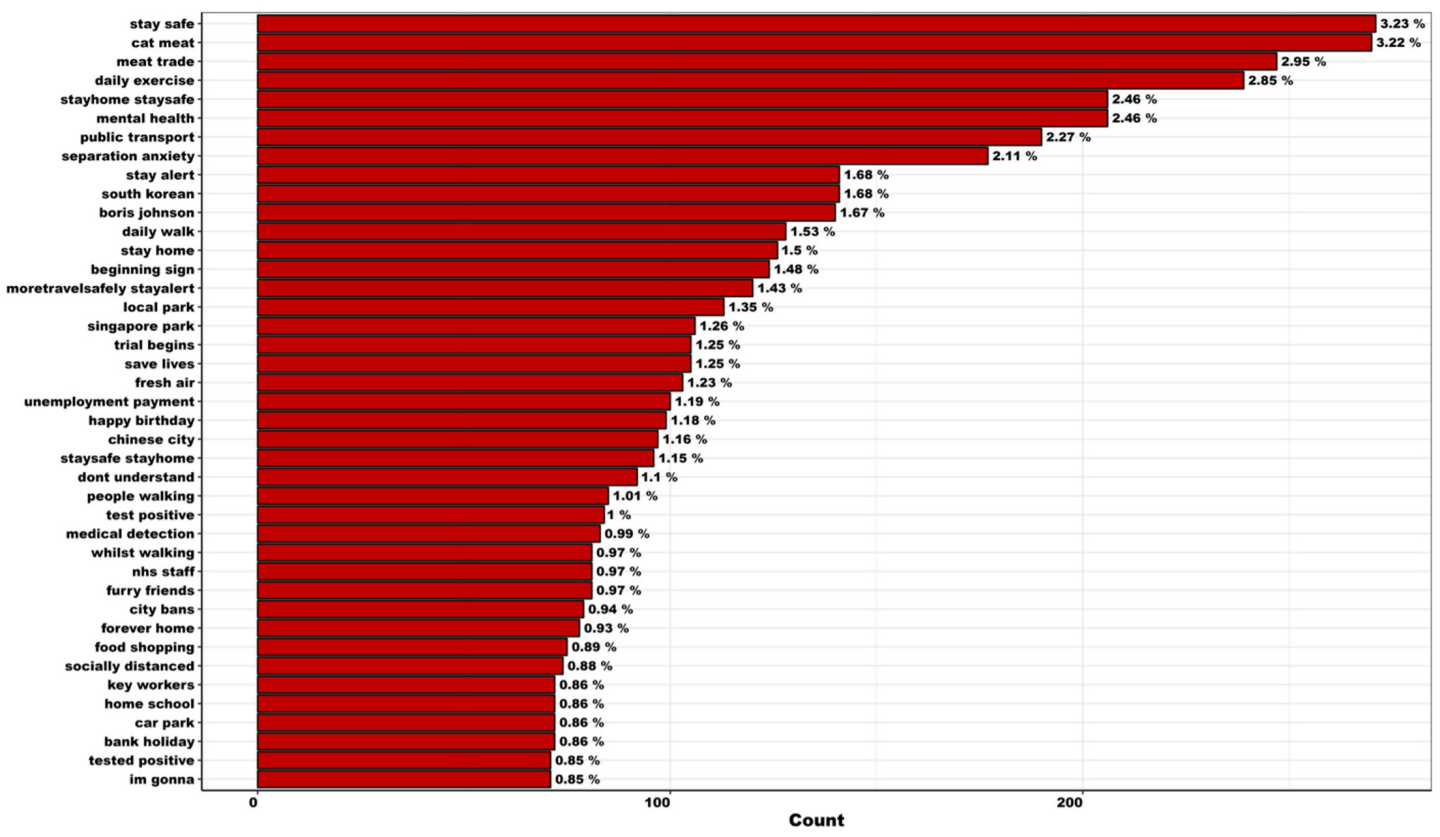
Most frequently used bigrams observed more than twice (to remove possible misspellings and/or inaccuracies) within tweets regarding canine-COVID topics in the UK and ROI, across the full period (March 27th–August 22nd, 2020; *n* = 8,383).

Between March 27th and August 22nd, 2020, 73,551 tweets regarding COVID-19 and dogs were collected from the UK and ROI. During data cleaning and manipulation, 8,300 duplicate tweets were removed and 4,163 were identified as irrelevant. Consequently, the final dataset included 61,088 unique tweets, posted from 42,403 Twitter accounts. Of these 61,088 unique tweets, 75.1% (*n* = 45,853) were original and 24.9% (*n* = 15,235) were replies. Our analysis indicated a rapid increase and a slow decline in the volume of social media conversations regarding COVID-19 and dogs (Supplementary Figure 2). Mean number of relevant words per tweet was 1.9 (SE = 0.01, range = 1–23) and daily mean number of tweets was 410.0 (SE = 21.0, range = 101–1,036). The mean number of tweets posted per account equated to 1.4 (SE = 0.01, range 1–391), and Twitter users applied 16,886 unique hashtags to original tweets. In total, 8,383 bigram combinations were observed more than twice (to remove possible misspellings and/or inaccuracies), throughout the full period, which are listed in Figure 2. The top 50 frequently used hashtags in original tweets are listed in Supplementary Figure 3.

### Variation between phases

Summary statistics regarding tweets per phase are listed in Supplementary Table 3. Across the full period, “walk” remained the most frequently used token. While “distance”, “home”, “like”, “love”, “work”, “help”, and “need” remained consistently well used (among others), the popularity of the initially well used hashtags “dogsduringlockdown” and “dogsoftwitter” diminished over time (Supplementary Figure 4). It is important to note that “help”, “like”, and “love” were frequently preceded by a negation term (e.g., “didn't”, “don't”, “no”, “not”, “wont”; Supplementary Figure 5). Consequently, these tokens should be considered with caution. Frequently used bigrams used throughout the full period, include meat trade and separation anxiety (Supplementary Figure 6).

**Figure 3:**
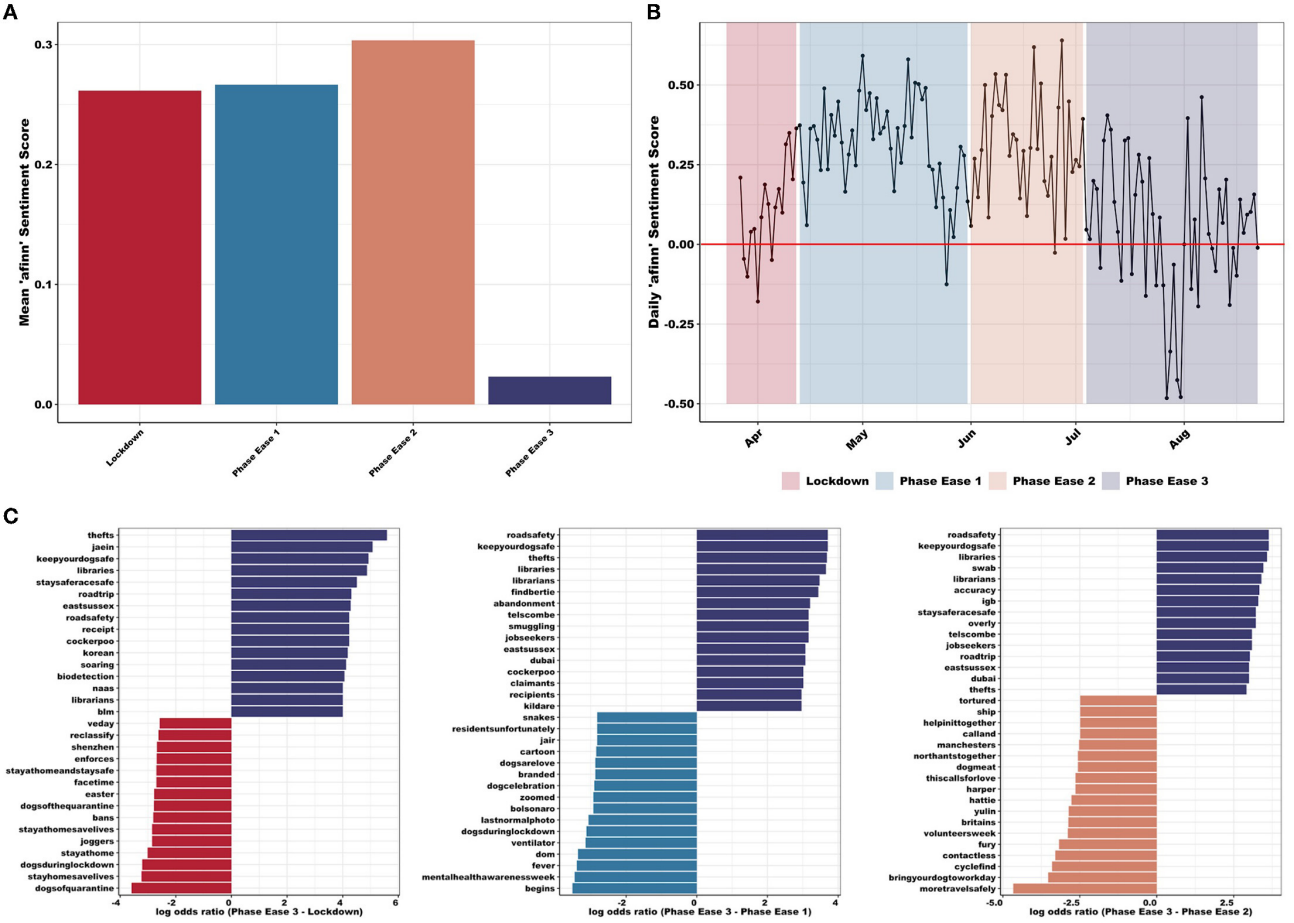
**(A)** Mean afinn sentiment scores differed significantly between the four phases (*F*_3, 78, 508_ = 44.4, *p* < 0.001) with PE3 exhibiting lower mean sentiment. **(B)** Daily afinn sentiment score (red line highlights zero, i.e., neutral and sentiment), presenting a decrease in sentiment during PE3 (i.e., increase in negative language use—regarding both intensity and frequency). Most negative days (and suggested potential reasons for these) include: 27th July (COVID-19 case was confirmed in cat in UK); 28th July (unknown reason); 30th July (increased local restrictions introduced in north-west England, no easing of further restrictions as planned elsewhere); 31st July (tighter lockdown restrictions introduced in north England and postponed easing); 5th August (tighter lockdown restrictions introduced to Aberdeen); and 14th August (UK experiencing heatwave and various news outlets reporting dogs left in hot cars). Most positive days (and suggested potential reasons for these) include: 1st May (news regarding COVID-19 testing and vaccines and Captain Tom Moore 's 100th birthday flypast); 14th May (news of dogs trained to protect wildlife in Africa); 8th June (announcements of lockdown restrictions easing including opening of hospitality and travel); 11th June (introduction of “support bubbles”); 19th June (large easing of Welsh lockdown rules); and 27th June (good weather). **(C)** Log odds ratios comparing word usage between PE3 (purple) and all other phases (LD: red, PE1: blue, PE2: pink): emphasizing the importance of “thefts”, “keepyourdogsafe”, “roadsafety”, “jobseekers”, “abandonment”, and “smuggling” in PE3 compared with word usage within LD (e.g., “joggers” and “bans”), PE1 (e.g., “begins” and “dogsarelove”), and PE2 (e.g., “cyclefind” and “dogmeat”).

Overall, the average sentiment score decreased significantly in PE3, with respect to *afinn* sentiment scores (Figures 3A, B). Mean *afinn* sentiment score differed significantly between the four phases (*F*_3, 78, 508_ = 44.4, *p* < 0.001; Figure 3A) with PE3 exhibiting lower mean sentiment score [mean (SE) = 0.02 (0.02)] than LD [mean (SE) = 0.26 (0.01); *p* < 0.001], PE1 [mean (SE) = 0.27 (0.02); *p* < 0.001], or PE2 [mean (SE) = 0.30 (0.02); *p* < 0.001]. Examination of daily sentiment scores across the full period identified the most negative days falling within PE3 (Figure 3B). PE3 also exhibited increased language related to “anger”, “sadness”, “fear”, and “negative” sentiment, while also showing a decrease in “joy” and “positivity” (*nrc* sentiment; Supplementary Figure 7). Furthermore, PE3 presented lower total cumulative and mean *bing* sentiment score (Supplementary Figure 8).

As PE3 exhibited a significantly lower mean sentiment score (Figure 3A), word frequencies were compared between PE3 and all others (LD, PE1, and PE2). Due to the large number of tweets and high frequency of common words, correlation between all three remained consistently high (Supplementary Figure 9). Words with similar high frequencies in both sets of texts included “people”, “home”, “walk”, and “dogsoftwitter”. PE3-LD comparison presents “abandonment” and “unemployment” commonly within PE3 tweets, while “stayhomesaveslives” and “selfisolation” are frequently found in LD [*r*_(11, 873)_ = 0.86, *p* < 0.001]. When comparing PE3-PE1, the latter commonly uses words such as “begins” and “lockdownlife”, whereas “staff” and “muzzle” are used more often in PE3 [*r*_(9, 030)_ = 0.88, *p* < 0.001]. During PE2, words such as “transport” and “cycle” are commonly located, whereas “thefts” and “hero” are more commonly found in PE3 [*r*_(8, 929)_ = 0.93, *p* < 0.001]. Words also extend to lower frequencies for PE3-LD comparison, which indicates that PE3 and LD use more similar wording than PE3-PE1 or PE3-PE2. These results are mirrored in Figure 3C where words from LD, PE1, and PE2 are compared to PE3. These results emphasize the importance of “thefts”, “keepyourdogsafe”, “roadsafety”, “jobseekers”, “abandonment”, and “smuggling” in PE3 compared with all other phases, which are also reflected in the TF-IDF analyses (Supplementary Figure 10).

### Variation between sectors

Across the full period, 386 “Personal” accounts, 104 “Press” accounts, 35 “State” accounts and 229 “Other” accounts contributed 9,259 tweets. Of these 754 sector-assigned accounts, 65 (i.e., 8.6%) were identified as potential bot accounts. However, these accounts were not removed from further analyses as verification required in-depth investigation. Of the 9,259 tweets assigned to the aforementioned sectors, 55.5% (*n* = 5,135) occurred during LD, 16.4% (*n* = 1,515) during PE1, 15.5% (*n* = 1,438) during PE2 and 12.6% (*n* = 1,171) during PE3 (Supplementary Figure 11). Mean number of relevant words per tweet was 2.6 (SE = 0.02, range = 1–19). Summary statistics regarding these tweets are listed in Supplementary Table 4.

**Figure 4:**
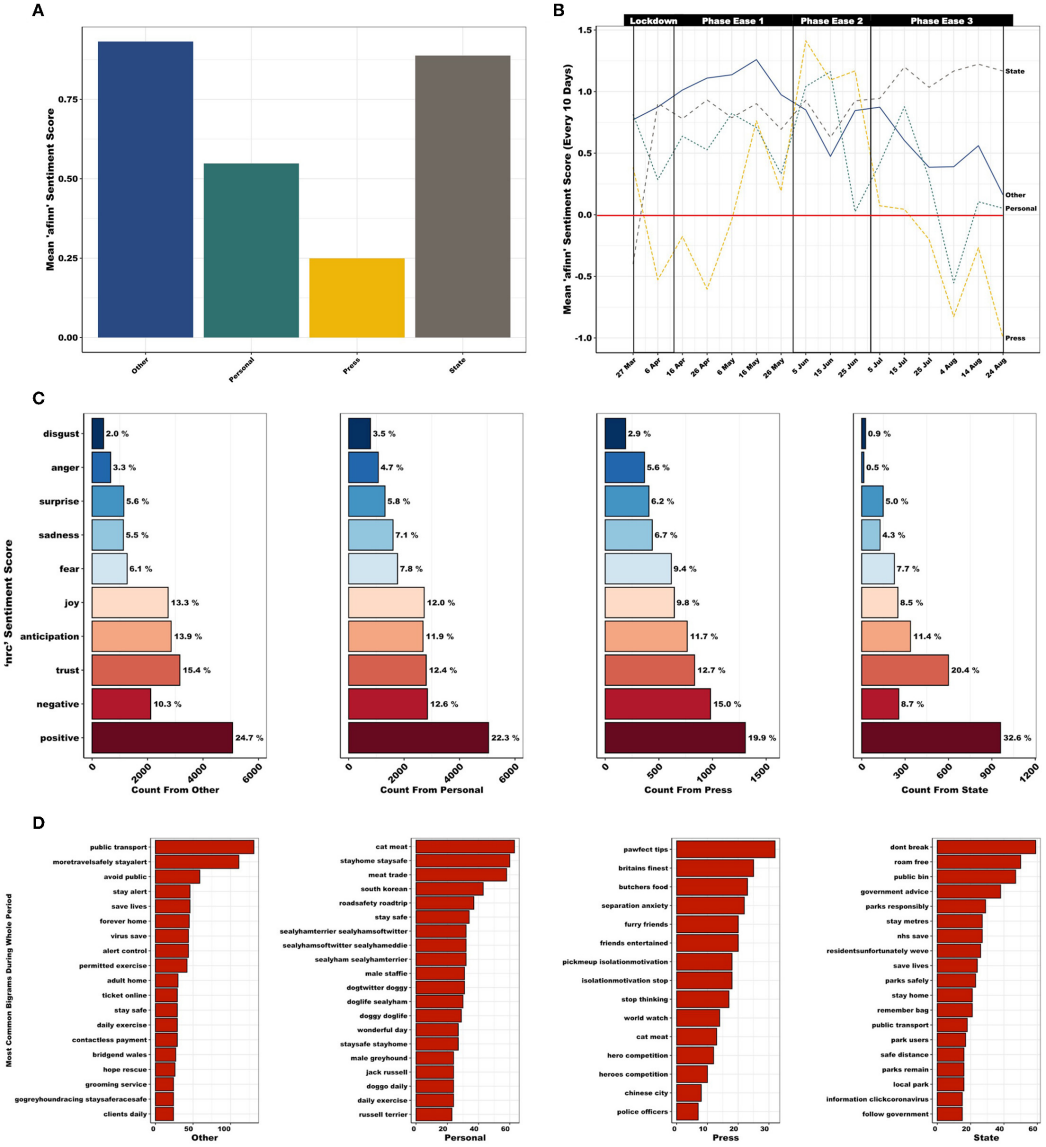
**(A)** Mean afinn sentiment scores, across the full period (March 27th–August 22nd, 2020), differed significantly between the four sectors (*F*_3, 11, 794_ = 52.2, *p* < 0.001) with Personal and Press accounts exhibiting lower mean sentiment. **(B)** Mean afinn sentiment score, every 10 days (red line highlights zero, i.e., neutral, sentiment). A decrease in sentiment is observed for Other, Personal, and Press accounts during PE3, whereas State presents a slight increase. **(C)** nrc sentiment scores (%), across the full period, exhibits variation in language use between the four sectors. **(D)** Most frequently used bigrams used between the four sectors, across the full period. “Other” accounts focused on travel/public transport (e.g., “public transport” and “moretravelsafely stayalert”), rules/safety (e.g., “avoid public” and “stay alert”) and the practicalities of lockdown (e.g., “ticket online” and “contactless payment”). Personal accounts focused on meat trade (e.g., “cat meat” and “meat trade”), travel/public transport (e.g., “roadsafety roadtrip”), location specific topics (e.g., “south korean”) and canine focused (e.g., “doggo daily” and “doggy doglife”). Press covers a wide variety of topics, including canine focused (“pawfect tips” and “separation anxiety”), mental health (e.g., “pickmeup isolationmotivation”), meat trade (e.g., “cat meat”) and location specific topics (e.g., “chinese city”). State accounts were particularly interested in rules/safety (e.g., “dont break” and “government advice”) and outdoor space (e.g., “roam free”, “public bin”, and “parks responsibly”).

Mean *afinn* sentiment score differed significantly between the four sectors (*F*_3, 11, 794_ = 52.2, *p* < 0.001; Figure 4A) with Press exhibiting lower mean sentiment score [mean (SE) = 0.25 (0.06)] than Other [mean (SE) = 0.93 (0.03); *p* < 0.001], Personal [mean (SE) = 0.55 (0.03); *p* < 0.001] and State [mean (SE) = 0.89 (0.05); *p* < 0.001]. Personal accounts also exhibited a lower mean sentiment score than Other (*p* < 0.001) and State (*p* < 0.001). Finer temporal examination of sentiment across the full period (i.e., mean sentiment score every 10 days; Figure 4B) shows that Other, Personal and Press decrease in sentiment during PE3, whereas State shows a slight increase. With regards to *nrc* sentiment (Figure 4C), Other accounts score highly for “joy” and “anticipation”, while scoring low for “fear”. Personal accounts score highly for “disgust” and “sadness”, while scoring low for “trust”. Press accounts score poorly for “joy” and “positive”, and score highly for “anger,” “fear”, and “negative”. State accounts score highly for “trust” and “positive”, and score poorly for “disgust”, “anger”, and “negative”. Furthermore, Press accounts presented a lower total cumulative and mean *bing* sentiment score (Supplementary Figure 12).

Throughout the full period, “walk” and “help” remain consistently well used across all sectors (Supplementary Figure 13). However, popular hashtags, e.g., “dogsduringlockdown” and “dogsoftwitter”, and more emotive language, e.g., “love” and “like” are only present within Other and Personal accounts (however, refer to Supplementary Figure 5 regarding negation issues). Press accounts frequently use terminology related to “pet” and “owner”, e.g., “train”, “warn”, “food”, and “tip”. Meanwhile State accounts refer to restrictions and public spaces often, e.g., “park”, “distanc-”, “bin”, “rule” etc. The most frequent bigrams per sector suggests that the main foci of conversations varied between sectors (Figure 4D). “Other” accounts focused on travel/public transport, rules/safety, and the practicalities of lockdown. Personal accounts focused on meat trade, travel/public transport, location specific and canine focused topics. Press covered a wide variety of topics, including canine focused, mental health, meat trade and location specific topics. State accounts were particularly interested in rules/safety and outdoor space.

**Figure 5:**
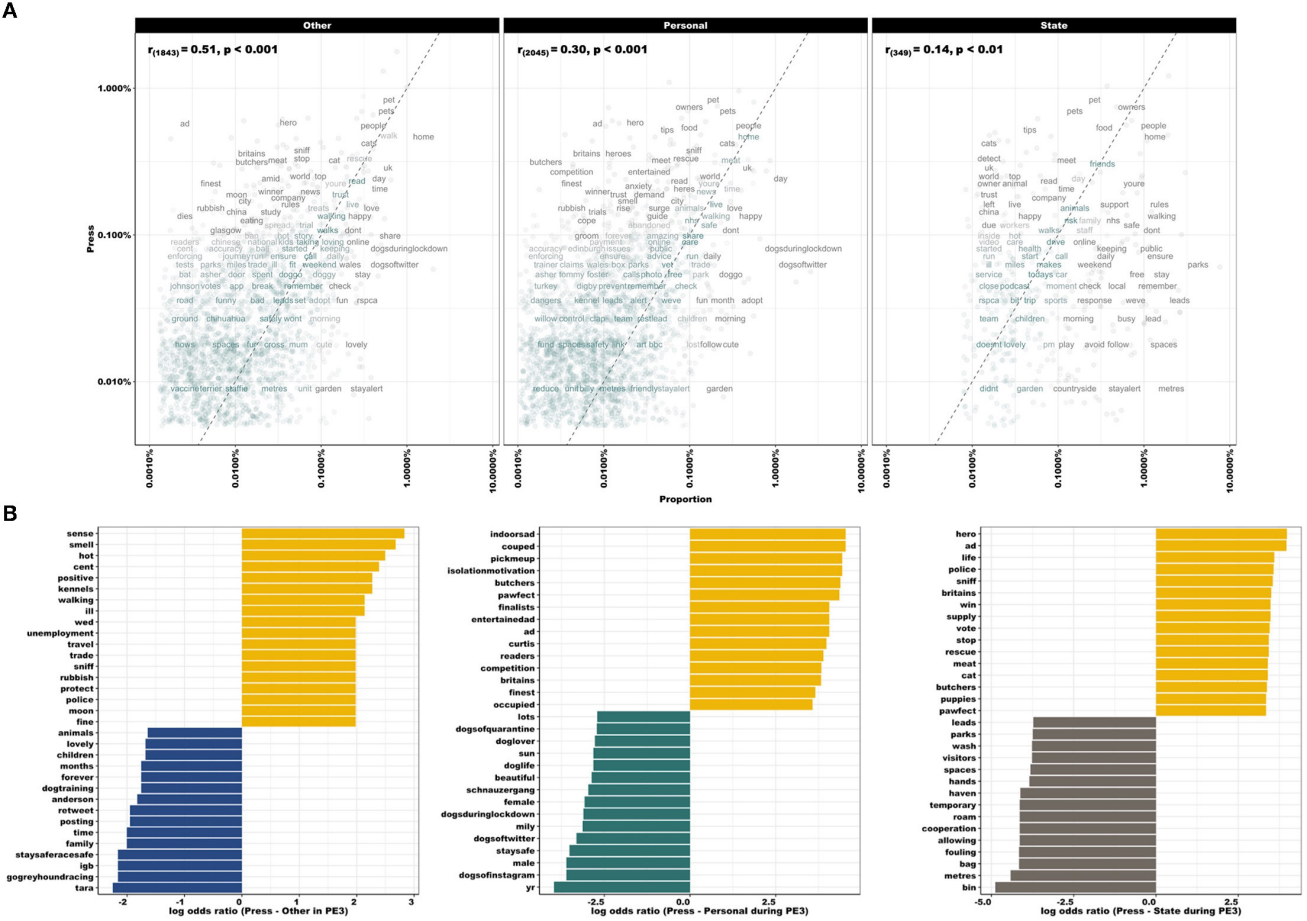
**(A)** Comparing word frequencies of Press with all other sectors (Other, Personal, and State), across the full period (March 27th–August 22nd, 2020). Pearson correlation coefficient noted in top left of panel. Words close to the zero-slope line have similar frequencies in both sets of texts, e.g., “people” and “home” are located at the upper frequency for all pairings. Words that are far from the line identify those that are found more in one set of texts than another. Empty space at low frequencies within Press-State panel indicates that these sectors use more dissimilar wording than Press-Other and Press-Personal (also represented by correlation coefficient). However, also note fewer data points in the Press-State panel. **(B)** Log odds ratios comparing word usage during PE3, between Press (yellow) and all other sectors (Other: blue, Personal: green, State: gray). Note increased use of language related to mental health, meat trade and employment, e.g., “indoorsad”, “couped”, “isolationmotivation”, “meat”, “trade”, and “unemployment”.

Figure 5A compares word frequencies of Press with all other disciplines (Other, Personal and State). In the Press-Other panel, “dies” and “finest” are found in Press tweets, while “lovely” and “share” are frequently found in Other [*r*_(1, 843)_ = 0.51, *p* < 0.001]. When comparing Press-Personal, the latter used words such as “cute” and “adopt”, whereas Press commonly used “hero” and “anxiety” [*r*_(2, 045)_ = 0.30, *p* < 0.001]. Within State accounts, words such as “park” and “spaces” were commonly found, whereas “cats” and “owner” were more commonly found in Press tweets [*r*_(349)_ = 0.14, *p* < 0.01]. Press-Other sectors were most highly correlated, whilst Press-State use more dissimilar wording than Press-Other and Press-Personal, indicated by the empty space at low frequencies within the Press-State panel. Log odds ratios comparing word usage during PE3 between Press and all other sectors (Figure 5B) emphasized the importance of “indoorsad”, “unemployment”, “meat”, “trade”, “couped”, and “isolationmotivation” within Press accounts, compared with word usage in Other (e.g., “gogreyhoundracing”, “family”, and “dogtraining”), Personal (e.g., “dogsofinstagram”, “beautiful”, and “doglover”) or State (e.g., “bin”, “fouling”, and “parks”) accounts.

### Cross correlation functions and lagged regressions

**Figure 6:**
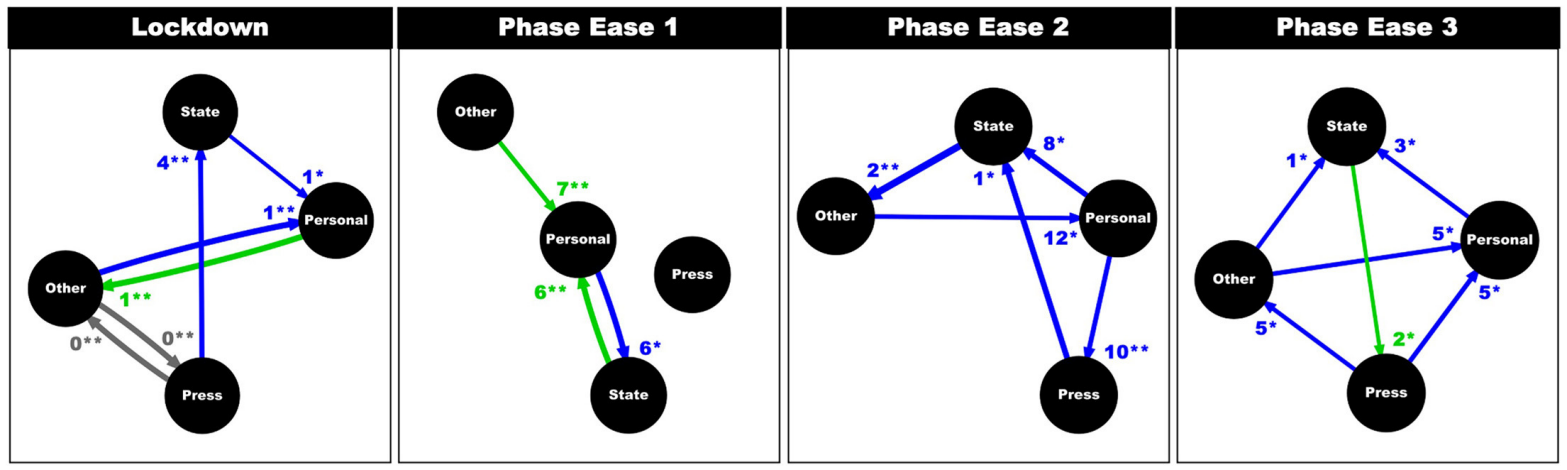
Results from cross correlation functions and lagged regressions, per phase. Each flowchart exhibits relationships between the four sectors, with regards to positive (green; above average sentiment in X is likely to lead to above average sentiment in Y), negative (blue; above average sentiment in X is likely to lead to below average sentiment in Y), or no (gray) correlation in mean sentiment. Arrows represent flow of influence and thickness of arrow represents strength of correlation. Lag (days) at which cross correlation is maximum is noted at arrowhead, along with statistical significance (*p* < 0.5*, p < 0.01**). Note that at the start of the pandemic (LD and PEl) Personal accounts were initially influenced by State and Other accounts (in both positive and negative directions). However, by PE3, Personal accounts were no longer correlated with State and Other accounts but Press and Other accounts.

During LD, above average sentiment (i.e., positive language use) from Other and State accounts correlated with below average sentiment (i.e., greater use of negative language) from Personal accounts, on the following day [CCF(−1) = 0.40, *p* > 0.01; CCF(−1) = 0.26, *p* = 0.05; Figure 6]. Simultaneously, above average sentiment in Personal accounts was followed by increased sentiment in Other accounts 1 day later [CCF(1) = 0.40; *p* < 0.01]. Negative language use (i.e., below average sentiment) from Press accounts correlated with an increase in positive language use (i.e., above average sentiment) by State accounts, 4 days later [CCF(−4) = 0.34, *p* < 0.01]. Press and Other exhibited similar sentiment on the same day [CCF(0) = 0.39; *p* < 0.01 for both].

During PE1, sentiment with Other and State was still associated with that of Personal accounts. However, during this phase, unlike above, these relationships were positive: above average sentiment in Other and State was followed by above average sentiment within Personal accounts 7 and 6 days later, respectively [CCF(7) = 0.30; *p* = 0.01; CCF(6) = 0.39, *p* < 0.01]. Additionally, negative language use (i.e., below average sentiment) in Personal accounts was associated with an increase of positive language (i.e., above average sentiment) within State accounts 6 days later [CCF(−6) = 0.39; *p* = 0.03].

During PE2, above average sentiment within Other accounts were likely to precede below average sentiment within Personal accounts 12 days later [CCF(−12) = 0.29, *p* = 0.02]. Below average sentiment in Personal accounts preceded increased sentiment in Press and State accounts 10 and 8 days later [CCF(−10) = 0.32; *p* < 0.01; CCF(−8) = 0.35; *p* = 0.02]. Below average sentiment score in Press was associated with above average sentiment score in State 1 day later [CCF(−1) = 0.38, *p* = 0.02], and below average sentiment score in State was associated with above average sentiment score in Other 2 days later [CCF(−2) = 0.46, *p* < 0.01].

During PE3, above average sentiment in Other and Press were likely to be followed by below average sentiment within Personal accounts 5 days later [CCF(−5) = 0.30; *p* = 0.03; CCF(−5) = 0.31; *p* = 0.02]. Below average sentiment in Personal accounts preceded above average sentiment scores within State accounts 3 days later [CCF(−3) = 0.28; *p* < 0.05]. Below average sentiment score in Press was associated with above average sentiment score in Other 5 days later [CCF(−5) = 0.28; *p* = 0.04]. Meanwhile, above average sentiment score in State was associated with above average sentiment score in Press 2 day later [CCF(2) = 0.26; *p* = 0.02], and above average sentiment score in Other was associated with below average sentiment score in State 1 day later [CCF(−1) = 0.28; *p* = 0.04].

Thus, during LD and PE1, Personal accounts were influenced by State and Other accounts, initially negatively (1 day later) and then positively (~1 week later), respectively. However, by PE3, whilst Personal accounts were still influenced by Other (5 days later), they were no longer influenced by State. Instead, sentiment displayed by Personal accounts was more closely aligned with Press accounts, 5 days later. Furthermore, Personal accounts were only positively influenced during PE1, by Other and State accounts (~1 week later). During all other phases (LD, PE2, and PE3), Personal accounts were negatively influenced by one or more sector. Finally, lag (days) at which cross correlation was maximum, seems to increase as time from the start date expands.

## Discussion

We present temporal and sector specific insights into online behavior and cohort opinion, obtained through the application of text mining and sentiment analysis. We focus on canine welfare during a pandemic as an example topic, to demonstrate the functionality and effectiveness of applying these tools to scraped Twitter data. This paper aims to serve as proof of concept for applying these computational tools to topics related to animal welfare, in the hope of encouraging the application of these methodologies within the sector. However, please note limitations of the above methodologies are discussed in Supplementary Note 1.

Gathering insight into public opinion, along with tracking and assessing main topics discussed online, aids our understanding of attitudes. Attitudes enable humans to determine, often very quickly, who to interact with, which products to purchase and/or which behaviors to engage in Sheeran et al. (66) and Maio and Olson (67). Given that human behavior shapes the welfare of our companion animals, due to the nature of the pet-owner relationship, it is important that we understand the public's attitude toward animals and their welfare. Due to this, a key interest of this paper was to highlight the capability of using Twitter data to identify topical foci of conversations regarding canine-COVID topics in the UK and ROI.

Within our dataset, tweets regularly mentioned positive tokens such as “love” and “together”. Several studies have identified dogs as being a source of purpose, routine, and entertainment for their owners during lockdown (25–27, 68). Positive implications of the pandemic have included owners reporting improved emotional bonds and/or more regular interaction with their pets (25, 69, 70). It is therefore unsurprising that people's focus turned toward this bond. Dogs are often depicted as providing their owners with comfort and feelings of relief during uncertain times, by offering companionship and interaction that would otherwise be lacking (23, 68, 69). This sense of companionship may have influenced the increase in web interest regarding adoption of cats and dogs during the early phase of the COVID-19 pandemic (23), which has been suggested to be sustainable for cats, but not dogs (71). This topic may be present within our dataset, due to the frequently used token “adopt” within Personal accounts, and “smuggling” within PE3 conversations, potentially referring to the increased demand for puppies and associated intensification of large-scale breeding and/or rise of illegal puppy smuggling.

While the pandemic provided a unique opportunity to build bonds between owner and pet, there is growing concern regarding the impact it may have had on canine welfare. For example, the most prominent token used throughout all phases was “walk”, along with associated bigrams: “local park”, “fresh air”, “whilst walking”, and “daily walk”. Due to lockdown restrictions and further guidelines (72–74), it is likely that there has been a change to daily routines, which may have altered the frequency, duration, and location that owners walk their dog. Research surveying dog owners found that for many, dog walking became a treasured experience, offering the opportunity to get outdoors, maintain some form of routine and improve wellbeing (25). Despite this fact, others have reported that during lockdown, dogs were typically walked less often, for less time daily and had fewer opportunities to interact with other dogs: thus, decreasing opportunities for enrichment, socialization, and cognitive stimulation (24, 75). Either in response to, or predicting negative fallout as a result of this, Press accounts frequently referred to terminology relating to these issues, e.g., “pet”, “owner”, “train”, “warn”, “food”, and “tip”. Furthermore, due to local restrictions, reports of high numbers of dog walkers flocking to local outdoor spaces boomed (76), potentially increasing the amount of dog fouling in these spaces. Dog fouling presents a significant public health concern and can reduce the mental and physical wellbeing of nearby residents (77, 78). Furthermore, it has been stated that “dog excrement can have a significant economic impact in terms of deterring inward investment and tourism” to an area (79). Given these prior reasons, and the fact that activities associated with dog waste collection and disposal can be financially restrictive for local authorities, it is unsurprising that State accounts frequently use terms related to dog fouling, e.g., “public bin”, “remember bag”, and “parks responsibly”, along with “bin”, “bag”, and “fouling”. This topic is especially noteworthy as access to green space during the pandemic has been linked to a wide range of mental health outcomes and has been suggested as an essential quality-of-life element (80, 81).

While there were several foci of conversation, two topics remained consistently prominent throughout the full period: meat trade and separation anxiety, particularly within Personal and Press accounts. Discussion of the meat trade mostly referred to international campaigns seeking world-wide bans of both dog and cat meat-trade, along with associated closures of wet markets. With regards to separation anxiety, as a result of lockdown measures, dogs are reported to have spent more time in the company of their owner (24). With the relaxation of these measures, resultant changes to dog management and time spent alone may have increased likelihood of longer-term welfare issues such as dogs displaying separation-related behaviors, e.g., excessive barking, aggression, and destructive behavior (24, 82). As behavioral problems are reported to be one of the main reasons for the relinquishment to shelters (83), there is growing concern that the pandemic has affected adoption and abandonment of dogs (23). This concern is mirrored within our data by the frequent use of “abandonment” during PE3, and “adopt” within Personal accounts. While we are yet to see the full impact of the pandemic on relinquishment rates, our findings, and those from previous studies highlight the importance of further research into crisis-driven changes in human–animal relationships.

During PE3, issues surrounding dog theft became prominent, e.g., “keepyourdogsafe” and “thefts”. Results of a BBC freedom of information request stated that five policing areas recorded a double-digit increase in the total number of dog thefts reported between January and July 2020, compared with the previous year (84). Furthermore, there were an estimated 2,000 incidents of dog theft reported in England and Wales in 2020 (85). This apparent increase in dog theft may be associated with the increasing demand for dogs during lockdown (23, 71). However, due to a lack of informative, comparable, and accessible datasets regarding dog thefts, analyzing spatiotemporal patterns, including incidence, remains very challenging (86). Future amendments to sentencing guidelines (85, 87) associated with the Theft Act (1968) (88) should consider adopting a standardized, centrally held data management system with a robust identifier for “pet theft”, allowing for a stronger spatial and temporal evidence base regarding the problem. The urgency of this topic, within the public domain, is clear within our results.

Hashtags can be viewed as topical markers, an indication of the tweet context or as the core topic expressed in the tweet. As such, researchers often assume that relevant populations consist of Twitter users who index their tweets with specific hashtags. Within this data, while the most frequently used hashtags “dogsduringlockdown” and “dogsoftwitter” appeared frequently within Personal and Other accounts, Press and State did not commonly utilize trending hashtags. Given the potential to improve one's “searchability” and attract a relevant audience by utilizing clear hashtags (89, 90), it may be prudent to suggest this to be a missed opportunity. However, while these hashtags were utilized frequently during LD and PE1, their popularity diminished with time, reflecting the fast-paced and evolving nature of Twitter. Romero et al. (91) stated that different categories of hashtags have different propagation patterns, introducing the distinction between “stickiness” and “persistence”: arguing that some classes of hashtag are more persistent than others. Our work shares these observations and highlights that delineating populations *via* hashtags may create analytical issues for researchers downstream, i.e., when tweets must be categorized as relevant vs. “noise”, as hashtag use are both sector and temporally biased.

Studies have reported that people living under lockdown measures are more prone to evolve various psychological symptoms, e.g., stress, depression, emotional fatigue, insomnia, and signs of post-traumatic anxiety (92). This may be reflected in the frequent use of terms relating to mental health throughout the full period, but especially during PE3, i.e., “indoorsad”, “couped”, and “isolationmotivation”. Thus, it is somewhat unsurprising that the psychological stress of the pandemic generated a stark negative response during PE3 within Personal, Press and Other accounts, represented by the rapid rise in negative sentiment, i.e., language related to “anger”, “sadness”, “fear”, and “negativity”. These results are supported by a previous study focusing on Chinese social media usage, which found an increase in negative emotions (i.e., depression and anxiety) and a decrease in positive emotions and life satisfaction when compared to pre COVID-19 times (93). In contrast, State accounts presented a slight increase in positive sentiment during PE3, frequently using language relating to “trust” and “positivity” and rarely using language relating to “disgust”, “anger”, “sadness”, or “negativity”. There are multiple factors that may influence variation in the language used by sectors online, e.g., target audience, communication direction flow, marketing strategies (reactive vs. proactive) and personal/corporation aims. However, the temporal differences in sentiment portrayal evident across groups suggests influence between groups and, potentially, emotional contagion, i.e., whereby user sentiment may be affected by others (94). The impact of emotional contagion may be considered positive (e.g., informed science communication) or negative (e.g., inaccurate information exchange leading to detrimental attitudes and diminished animal welfare). We examined the influence of one sector upon another and suggested the “electronic word of mouth” time delay that may exist between two sectors. Early in the pandemic (LD and PE1) Personal accounts appeared to be initially influenced by State and Other accounts. However, by PE3, Personal accounts were influenced by Press and Other accounts. Consequently, there was evidence of a shift in sector influence on public perception (at least regarding canine-COVID topics). These results suggest that the most influential sources, toward the public, may change over time: in this case State becoming less influential and Press more. This shift in public responsiveness to government messaging during times of crises has been previously suggested (95, 96). Additionally, the number of days taken to see change in sentiment within the affected sector may vary over time (in this case study it appeared to increase). Thus, influence between sectors may be more rapidly evident closer to the original starting point.

Effective and temporally accurate data is essential to the successful management and completion of any corporation or charity aims. Whilst these methods initially require computational infrastructure, knowledge and training, the sourcing of data is rapid, robust, rich, and inexpensive, especially in comparison to alternative methods aiming to ascertain attitudes, such as focus groups and online questionnaires. Given the real-time streaming nature of Twitter, ability to search archives for relevant topics, open access statistical products, free data visualization platforms, and online help communities, the methods described here provide a unique, yet untapped, tool for the animal welfare sector. Furthermore, while keeping pace with the online community may prove challenging, doing so may be beneficial to multiple stakeholders, as it should increase their online presence, embed them within pertinent conversations, increase their likelihood of remaining relevant and provide insights into online behavior and cohort opinion.

Our findings do not elucidate the role that sectors have on public perception of specific canine-COVID topics, we simply highlight that the information conveyed, and language used by sectors can have an impact on public perception. Sector influence may guide public attitudes toward animal welfare, which may, in turn, influence behavior (94). While this could directly impact upon human-animal interactions, both negatively and positively: it could also indirectly affect animal welfare by altering support toward specific topics, e.g., political causes. This highlights the importance of sector responsibility regarding appropriate and accurate messaging. As sectors were found to display varying sentiment, it is paramount that we continue to examine the influence of certain groups on public opinion. We suggest future studies elucidate opinion and sentiment surrounding specific animal welfare topics, potentially before and after behavioral intervention campaigns, to better inform the development of proactive response strategies.

## Data availability statement

The datasets presented in this article are not readily available due to Twitter's Developer Agreement. Requests to access the datasets should be directed to kirsten.mcmillan@dogstrut.org.uk.

## Ethics statement

Ethical approval for this study was granted by Dogs Trust Ethical Review Board (Reference Number: ERB036). Written informed consent from the participants' legal guardian/next of kin was not required to participate in this study in accordance with the national legislation and the institutional requirements.

## Author contributions

RC collected the data. KM designed methodology, analyzed the data, and led the writing of the manuscript. RC, KM, and KA conceived the ideas, contributed critically to the drafts, and gave final approval for publication. All authors contributed to the article and approved the submitted version.
