# Supplementary material for: Pooches on a platform: Text mining twitter for sector perceptions of dogs during a global pandemic
Source: Front Vet Sci. 2023 Mar 1;10:1074542. doi: 10.3389/fvets.2023.1074542 (PMC10014727; doi:10.3389/fvets.2023.1074542)
Supplement: Supplementary file 1 [file Data_Sheet_1.docx]

***Table 1:*** *Number of tweets retrieved which include specific search terms (including main text and hashtags). This is based on the initial scraping of data (n = 75,822 tweets), thus predates de-duplication, relevance check and subsetting to date range.*

| **SEARCH TERM (includes HASHTAG)** | **TWEETS RETRIEVED** | |
| --- | --- | --- |
|  | **N** | **%** |
| **TERM 1:** Words related to COVID-19 | | |
| lockdown/lock down | 34,569 | 45.6 |
| covid/co vid/coronavirus/c19/c 19 | 19,854 | 26.2 |
| walk/exerci- (includes walking/exercise/exercising etc.) | 16,018 | 21.1 |
| distanc- (includes distance/distancing/2 metre/two metre/2 meter/two meter etc.) | 12,768 | 16.8 |
| pandemic | 4,375 | 5.8 |
| isolat- (includes isolate/isolating/isolation etc.) | 4,308 | 5.7 |
| quarantin- (includes quarantine/quarantining etc.) | 3,591 | 4.7 |
| stay home/stayhome/stay at home/stayathome | 3,283 | 4.3 |
| work from home/workfromhome/working from home/ workingfromhome/WFH | 2,569 | 3.4 |
| stayalert/stay alert | 1,511 | 2.0 |
| home school/homeschool/home schooling/ homeschooling | 973 | 1.3 |
| furlough | 567 | 0.7 |
| cowork/co work (includes coworker/co working etc.) | 278 | 0.4 |
| **TERM 2:** Words related to dog(s) | | |
| dog- (includes dogs/doggo/doggy/doggies etc.) | 65,180 | 86.0 |
| pup (includes puppy/puppies etc.) | 6,786 | 8.9 |
| pooch (includes pooch/pooches etc.) | 1,053 | 1.4 |
| furbab- (includes furbaby/furbabys/furbabies etc.) | 245 | 0.3 |

***Table 2:*** *4,172 pre-defined terms used to check for relevance of tweets. List collated from previous Dogs Trust research projects^98^.*

| aanatolian | cattle swedish vallhund | estonian hound | hungarianvizsla | mioriticsheep | pyreneansheeplonghaired | sihpoo |
| --- | --- | --- | --- | --- | --- | --- |
| aaskan inuit | cattleaustralian | estonianhound | hungarianwater | mippto | pyreneansheepsmoothfaced | sihtzu |
| aaskaninuit | cattleflandersbouvier | estrela | hungarianwirehair | mite | pyreneanshepherd | silky |
| abruzzenhund | cattlegreatswiss | estrela mountain | hungarianwirehaird | mititiar | rabbit long haired | simaku |
| abyssinian | cattleswedishvallhund | estrelamountain | hungarianwirehaired | mix breed | rabbit smooth haired | situz |
| adopt | caucasian mountain | euasier | hungarianwirehairedvizsla | mixbreed | rabbitlonghaired | sitz shu |
| affenpin | caucasian ovtcharka | eurasian | hungr vizsla | mixed breed | rabbitsmoothhaired | sitzshu |
| affenpincher | caucasian sheep | eurasier | hungrvizsla | mixedbreed | rafeiro do alentejo | skipperke |
| afghan hound | caucasian shep | eurorussel | huntaway | moghrebi levrier marocain | rafeiro of alentejo | sloughi |
| afghanhound | caucasian shepard | faude britt | hunterway | moghrebilevriermarocain | rafeirodoalentejo | slovak rough haired |
| african boerboul | caucasian sheperd | faudebritt | hunting hound | mogrel | rafeiroofalentejo | slovakian chuvach |
| african bull | caucasian shepherd | fauve | huntinghound | molosser | rampur | slovakian hound |
| african hairless | caucasianmountain | fauve bretagn | huskamude | mongel | rastreador brasileiro | slovakian rough |
| african sand | caucasianovtcharka | fauve de bretagne | huskamut | mongr | rastreadorbrasileiro | slovakian rough haired |
| africanboerboul | caucasiansheep | fauve de bretagne griffon | huskamute | mongral | ratara | slovakianchuvach |
| africanbull | caucasianshep | fauvebretagn | huskey | mongrel | ratonero | slovakianhound |
| africanhairless | caucasianshepard | fauvedebretagne | huskidor | mongrol | ratonero bodeguero andaluz | slovakianrough |
| africansand | caucasiansheperd | fauvedebretagnegriffon | huskie | montenegrin mountain hound | ratonero mallorquin | slovakianroughhaired |
| agility | caucasianshepherd | fauves de bretagne | huskimute | montenegrinmountainhound | ratonero mallorquina | slovakroughhaired |
| aidi | cav k charles | fauvesdebretagne | husky | moodle | ratonerobodegueroandaluz | slovensky cuvac |
| airedal | cav king charles | feist | hybrid | moonflower | ratoneromallorquin | slovenskycuvac |
| airedale | cav king charlies | fench bull | hygen hound | morkee | ratoneromallorquina | sluki |
| akbash | cav spaniel | fenchbull | hygenhound | morkie | ratter | smalandsstovare |
| akita | cava poo | fenlander | hygenhund | morky | ratter prague prazsky krysavik | smooth coat |
| akkita | cavachion | fenyesi | i m unsure | moscow longhaired | ratterpragueprazskykrysavik | smooth hr |
| al co calato | cavachon | feral | iberian pedenco | moscow short haired | raystede | smoothcoat |
| alangu indian | cavachoun | fido | iberianpedenco | moscow vodolaz | red fox | smoothhr |
| alanguindian | cavadoodle | field setter | ibizan hound | moscowlonghaired | red heeler | smoushond |
| alano espanol | cavador | field spaniel | ibizan podenco | moscowshorthaired | red setter | snap |
| alanoespanol | cavailer | fieldsetter | ibizan warren hound | moscowvodolaz | red white | snarl |
| alapaha blood | cavailer kings charles | fieldspaniel | ibizanhound | mountain bernese | redfox | snauzer |
| alapaha blue | cavailerkingscharles | fila brasileiro | ibizanpodenco | mountain caucasian | redheeler | snchauzer |
| alapahablood | cavajack | filabrasileiro | ibizanwarrenhound | mountain cur | redsetter | sniff |
| alapahablue | cavalchon | finiish laphund | ibizian hound | mountain estrela | redwhite | snoodle |
| alascan malamut | cavalier | finiishlaphund | ibizian hunting | mountain greater swiss | rehome | snouser |
| alascanmalamut | cavalier kc | finish laphund | ibizianhound | mountain hound | rehoming | socialisation |
| alaskan malamute | cavalier kc spaniel | finish luphund | ibizianhunting | mountain portuguese | rerrier | soft coat |
| alaskan malaumute | cavalier king | finishlaphund | icelandic sheep | mountain pyrenean | rescue | soft coat wheaten |
| alaskanmalamute | cavalier king charles | finishluphund | icelandicsheep | mountain swiss | retreiver | soft coated weaten |
| alaskanmalaumute | cavalierkc | finnish hound | illyrian mountain | mountain view cur | retrietver | soft coated wheaten |
| alaskin malamute | cavalierkcspaniel | finnish laphapp | illyrian sheep | mountainbernese | retriever | soft coatedwheaten |
| alaskinmalamute | cavalierking | finnish laphound | illyrianmountain | mountaincaucasian | retriever chesapeake | softcoat |
| alastian | cavalierkingcharles | finnish laphund | illyriansheep | mountaincur | retriever chesapeake bay | softcoatedweaten |
| alaunt | cavalierpoo | finnish lapphund | imaal | mountainestrela | retriever curly coat | softcoatedwheaten |
| alcocalato | cavaliet | finnish spitz | imperial chinese | mountaingreaterswiss | retriever curly coated | softcoatwheaten |
| alear | cavalir | finnishhound | imperialchinese | mountainhound | retriever flat coat | solvakian wolf |
| alentejo | cavallier | finnishlaphapp | inuit american | mountainportuguese | retriever flat coated | solvakianwolf |
| allerca | cavapo | finnishlaphound | inuit american eskimo | mountainpyrenean | retriever golden | sooth hair |
| alopekis | cavapoo | finnishlaphund | inuit canadian eskimo | mountainswiss | retriever mountain | soothhair |
| alsatian | cavapoochan | finnishlapphund | inuit northern eskimo | mountainviewcur | retriever nova scotia | sorkie |
| alsation | cavapoochon | finnishspitz | inuitamerican | mouth cur | retriever nova scotia duck tolling | sottie |
| also apso | cavapooh | flanders cattle | inuitamericaneskimo | mouthcur | retriever novascotia | sottish |
| alsoapso | cavapooo | flanderscattle | inuitcanadianeskimo | moyen | retrieverchesapeake | south african boerboel |
| alstation | cavapooschon | flat coat | inuitnortherneskimo | mstif du | retrieverchesapeakebay | southafricanboerboel |
| am bull | cavapooshon | flat coat lab | irish balla | mstifdu | retrievercurlycoat | spaiel |
| ambull | cavapuchounon | flat coat retriever | irish blue | mstiff bordeaux | retrievercurlycoated | spainel |
| ameerican bull | cavaschon | flat coat rev | irish bull | mstiffbordeaux | retrieverflatcoat | spainiel |
| ameericanbull | cavashom | flat coated | irish glen of imaal | mucuchies | retrieverflatcoated | spanador |
| ameican bull | cavashon | flat coated retreiver | irish irish staffor | mudi | retrievergolden | spanal |
| ameicanbull | cavashun | flat coated retriever | irish irish staffordshire bull | mulshi | retrieverlabrador | spanial |
| amer bull | cavasion | flatcoat | irish red | multi poo | retrievermountain | spaniel |
| amerbull | cavatzu | flatcoat retriever | irish red and white | multipoo | retrievernovascotia | spaniel american |
| amercan bull | cavcachon | flatcoat spaniel | irish red and white setter | mungral | retrievernovascotiaducktolling | spaniel american ckr |
| amercanbull | cavelier | flatcoated | irish red setter | mungrel | retriver | spaniel american water |
| american bange | cavellier king charles | flatcoated retriever | irish setor | munster lander | retriver curly coated | spaniel cavalier |
| american bull | cavellierkingcharles | flatcoatedretreiver | irish setter | munsterlander | retrivercurlycoated | spaniel cs |
| american cockapoo | caverpom | flatcoatedretriever | irish water spaniel | munsterlander large | rhodesian | spaniel english |
| american coonhound | caverpoo | flatcoatlab | irish wheaten | munsterlanderlarge | rhodesian ridge | spaniel english springer |
| american eskimo | cavestie | flatcoatretriever | irish wold hound | musterlander | rhodesian ridgeback | spaniel field |
| american hairless | cavi | flatcoatrev | irish wolf hound | mutlipoo | rhodesianridge | spaniel irish water |
| american mosely | cavi chon | flatcoatspaniel | irish wolfhound | mutt | rhodesianridgeback | spaniel japanese |
| american shepherd | cavichion | flea | irishballa | muzzle | rich back | spaniel king charles |
| american staff | cavichon | floodle | irishblue | napolitan bull | richback | spaniel king charles kcs |
| americanbange | cavie | floof | irishbull | napolitanbull | ridge back | spaniel king charles spaniel |
| americanbull | caviechon | fopaulistinha | irishglenofimaal | neaplolitan | ridgeback | spaniel picardy |
| americancockapoo | caviepoo | forest hound | irishirishstaffor | neapolitan | ridgeback rhodesian | spaniel russian |
| americancoonhound | caviler | foresthound | irishirishstaffordshirebull | neopolian | ridgebackrhodesian | spaniel sussex |
| americaneskimo | cavilier | foretriever | irishred | neopolitan | rodesian | spaniel tibetan |
| americanhairless | cavipoo | forrest hound | irishredandwhite | neopolitan italian | rodesian ridgback | spanielamerican |
| americanmosely | cavischon | forresthound | irishredandwhitesetter | neopolitan mastive | rodesian ridgeback | spanielamericanckr |
| americanshepherd | cavish | four legged | irishredsetter | neopolitan napolitino | rodesianridgback | spanielamericanwater |
| americanstaff | cavishon | fourlegged | irishsetor | neopolitan ridgeback | rodesianridgeback | spanielcavalierk |
| amstraf | cavision | foxhound | irishsetter | neopolitanitalian | rodisian ridgeback | spanielcs |
| anatolian | cavkcharles | foxsmooth | irishwaterspaniel | neopolitanmastive | rodisianridgeback | spanielenglish |
| andalusian mouse | cavkingcharles | foxwire | irishwheaten | neopolitannapolitino | romagna lagotto | spanielenglishspringer |
| andalusianmouse | cavkingcharlies | french basset | irishwoldhound | neopolitanridgeback | romagnalagotto | spanielfield |
| anglos francai grand | cavli poo | french bassett | irishwolfhound | neuter | romagnolo | spanielirishwater |
| anglos francais de moyenne venerie | cavlipoo | french briard | istrian coarse haired hound | new zealand heading | romanian bucovina shepherd | spanieljapanese |
| anglos francaises | cavo bichon | french brittany | istrian hound | new zealand huntaway | romanian bukovina sheep | spanielkingcharles |
| anglosfrancaigrand | cavobichon | french bull | istrian short haired hound | new zealand hunter | romanian carpathian shepherd | spanielkingcharleskcs |
| anglosfrancaisdemoyennevenerie | cavoodle | french bulldog | istriancoarsehairedhound | new zealand hunterway | romanian mioritic shepherd | spanielkingcharlesspaniel |
| anglosfrancaises | cavopoo | french english | istrianhound | new zealand sheep | romanian rescue | spanielpicardy |
| apensell mountain | cavschon | french english bull | istrianshorthairedhound | newfie | romanian sheep | spanielrussian |
| apensellmountain | cavshon | french griffin | italian bracco | newfie leon shepherd | romanian shephard | spanielsussex |
| appenzell cattle | cavspaniel | french guard | italian bracco italiano | newfieleonshepherd | romanian shepherd | spanieltibetan |
| appenzell mountain | cavvie | french hound | italian bull | newfoundland | romanian street | spanielwelshspringer |
| appenzellcattle | cavvy | french hunting hound | italian cane corsa | newzealand sheep | romanianbucovinashepherd | spanish alano |
| appenzeller | ccker | french korthals | italian cane corso | newzealandheading | romanianbukovinasheep | spanish bull |
| appenzellmountain | central asia shepherd | french neopolitan | italian corsa | newzealandhuntaway | romaniancarpathianshepherd | spanish galgo |
| apsochi | central asian ovcharka | french pointing | italian grey hound | newzealandhunter | romanianmioriticshepherd | spanish hound |
| aquita | central asian ovtcharka | french pointing gascogne | italian hound | newzealandhunterway | romanianrescue | spanish hunting |
| arab bull | central asian sheep | french pointing pyrenean | italian hunter | newzealandsheep | romaniansheep | spanish mastin |
| arabbull | central asian sheepd | french sheep | italian lagotto | nichol frieze | romanianshephard | spanish mastine |
| arabian sloughi | central asian shepherd | french tricolour hound | italian lurcher | nicholfrieze | romanianshepherd | spanish mastiv |
| arabiansloughi | centralasianovcharka | french water | italian mastff | niese | romanianstreet | spanish pedenco |
| aredale | centralasianovtcharka | frenchbasset | italian piccolo levriero italiano | nivernais griffon | roottweiler | spanish perdiguero |
| arieg | centralasiansheep | frenchbassett | italian rough haired segugio | nivernaisgriffon | rot weiler | spanish pidenco |
| armant | centralasiansheepd | frenchbriard | italian segugio | nizinny valee | rotti | spanish podenco |
| artesian norman | centralasianshepherd | frenchbrittany | italian setter | nizinnyvalee | rottie | spanish pudenka |
| artesiannorman | centralasiashepherd | frenchbull | italian sheep | norbottenspets | rotttweiler | spanish sheep |
| artesien norman | cesky | frenchbulldog | italian shepard | norbrook | rottweile | spanish street |
| artesiennorman | chahuahua | frenchenglish | italian sighthound | nordic spitz | rottweiler | spanish water |
| arubian cunucu | chaiaua | frenchenglishbull | italian spinone | nordicspitz | rottweiller | spanish water spaniel |
| arubiancunucu | chalker | frenchgriffin | italian spinoni | norfolk terr | rottweller | spanishalano |
| aryan molossus | chalkie | frenchguard | italian volpino | norfolkterr | rottwieller | spanishbull |
| aryanmolossus | chalky | frenchhound | italianbracco | norrbottenspitz | rottwwieler | spanishgalgo |
| atlas mountain | charles cavalier | frenchhuntinghound | italianbraccoitaliano | norsk lundehund | rotty | spanishhound |
| atlas schaferhund | charles spaniel | frenchi | italianbull | norsk lundhehund | rotty lab | spanishhunting |
| atlasmountain | charlescavalier | frenchie | italiancanecorsa | norsklundehund | rotty staff | spanishmastin |
| atlasschaferhund | charlesspaniel | frenchkorthals | italiancanecorso | norsklundhehund | rottylab | spanishmastine |
| australian bull | charoulet | frenchneopolitan | italiancorsa | north american shepherd | rottystaff | spanishmastiv |
| australian cattle | charplaninatz | frenchpointing | italiangreyhound | northamericanshepherd | rotweiler | spanishpedenco |
| australian cobber | chauhau | frenchpointinggascogne | italianhound | northern inuit | rotweiler bull | spanishperdiguero |
| australian kelpie | chavachon | frenchpointingpyrenean | italianhunter | northerninuit | rotweilerbull | spanishpidenco |
| australian koolie | chavashon | frenchsheep | italianlagotto | norwegian buhund | rotweiller | spanishpodenco |
| australian labra | chavelon | frenchtricolourhound | italianlurcher | norwegian elkhound | rotwieler | spanishpudenka |
| australian shepherd | chaverpoo | frenchwater | italianmastff | norwegian elkound | rough belgian griffon | spanishsheep |
| australian silky | chavicon | friesland | italiano macellaio | norwegian hound | rough coat | spanishstreet |
| australianbull | chavishon | frisian water | italianomacellaio | norwegian lundehund | rough coated lakelan | spanishwater |
| australiancattle | cheasapeake bay retriever | frisianwater | italianpiccololevrieroitaliano | norwegian sheep | rough haired | spanishwaterspaniel |
| australiancobber | cheasapeakebayretriever | furbab | italianroughhairedsegugio | norwegianbuhund | rough pulli | spay |
| australiankelpie | chesapeake bay ret | furgoodfridays | italiansegugio | norwegianelkhound | roughbelgiangriffon | spingador |
| australiankoolie | chesapeake bay retiever | furry | italiansetter | norwegianelkound | roughcoat | spinger |
| australianlabra | chesapeake bay retriever | g retriever | italiansheep | norwegianhound | roughcoatedlakelan | spinger span |
| australianshepherd | chesapeake bay retriver | galgo | italianshepard | norwegianlundehund | roughhaired | spinger spaniel |
| australiansilky | chesapeakebayret | galgo espanol | italiansighthound | norwegiansheep | roughpulli | spingerdor |
| australn silky | chesapeakebayretiever | galgoespanol | italianspinone | norweigan buhund | round worm | spingerspan |
| australnsilky | chesapeakebayretriever | galiar | italianspinoni | norweiganbuhund | roundworm | spingerspaniel |
| auvergne | chesapeakebayretriver | gascogne | italianvolpino | nova scotia duck toller retriever | rspca | spinone |
| azawa | chesky | gascon | j r t | nova scotia duck tolling retriever | rtriever | spinone italiano |
| balinese | chewawa | gascon saintongeois | j russell | nova scotia retreiver | rtriver | spinoneitaliano |
| balkan hound | chi hua hua | gasconsaintongeois | j russell border | nova scotia retriever | rttie | spirnger |
| balkanhound | chiahua | gazelle hound arabian | jachuahua | novascotia duck tolling retrie | russell | spitz |
| barbet | chiahuahua | gazellehoundarabian | jacj russell | novascotia duck tolling retriever | russell b t | spitz finnish |
| bark | chiauha | geman shep | jacj russell border | novascotiaducktollerretriever | russellbt | spitz generic |
| barshar | chiauhoua | gemanshep | jacjrussell | novascotiaducktollingretrie | russian bear | spitz german |
| basenj | chiauhua | germ shep | jacjrussellborder | novascotiaducktollingretriever | russian bolonka | spitz german giant |
| bassedor | chichaua | german deutsche | jack a doddle | novascotiaretreiver | russian european laika | spitz japanese |
| basset | chichu | german hunt | jack a poo | novascotiaretriever | russian hound | spitz mittel |
| basset griffon vendeen | chichua | german hunter | jack a wawa | nz huntaway | russian ovtcharka | spitz nordic |
| basset hound | chichuahua | german hunting | jack r | nzhuntaway | russian sheep | spitz pom |
| bassetgriffonvendeen | chichuaua | german long haired | jack rus | old danish pointing | russian shepherd | spitz powder puff |
| bassethound | chichucha | german longhaired | jack ruseel | old english bull | russian spaniel | spitzfinnish |
| bassett | chien d artois | german rough haired | jack rusell | old english sheep | russian tsvetnaya bolonka | spitzgeneric |
| bassett hound | chien de montagne des | german rough haired pointing | jack russ | old english tyme bullgie | russian unicorn | spitzgerman |
| bassetthound | chien de pays | german roughhaired | jack russel | old spanish bull | russian wolfhound | spitzgermangiant |
| bassnji | chien de saint hubert | german sheep | jack russell | old thyme bull | russianbear | spitzjapanese |
| battersea | chien de trait | german sheeperd | jack russer | old time bull | russianblack | spitzmittel |
| bavarian mountain | chien francais blanc et noir | german shep | jack russerl | old time dorset bull | russianbolonka | spitznordic |
| bavarianmountain | chien loup | german shepard | jack russle | old time english bull | russianeuropeanlaika | spitzpom |
| bazenji | chiendartois | german shepeard | jack russsell | old time farm shepherd | russianhound | spitzpowderpuff |
| bchon frise | chiendemontagnedes | german sheperd | jack russuel | old tyme bull | russianovtcharka | spniel |
| bchonfrise | chiendepays | german shephard | jack russuell | old tyme bullgie | russiansheep | spocker |
| beachion | chiendesainthubert | german shepheard | jacka | old tyne bull | russianshepherd | spocker spaniel |
| beagador | chiendetrait | german shepher | jacka poo | old victorian bull | russianspaniel | spockerspaniel |
| beagal | chienfrancaisblancetnoir | german shepherd | jackabea | olddanishbull | russiantsvetnayabolonka | sporting lucas |
| beagel | chienloup | german sheppard | jackadoddle | olddanishpointing | russianunicorn | sportinglucas |
| beagle | chiens francaises | german shepperd | jackahuahua | olde boston bullge | russianwolfhound | spr spaniel |
| beaglier | chiensfrancaises | german shepphard | jackal | olde english bull | russiky | spreagle |
| bearded col | chih | german sherpard | jackapoo | olde english bullge | russn black | spriner |
| beardedcol | chiha | german sherperd | jackas | olde tyme bullge | russnblack | spriner spaniel |
| beauceron | chihauha | german sherpherd | jackawawa | olde tyme english | russo european laika | sprinerspaniel |
| becion | chihauhau | german shhephard | jackawuwhu | olde victorian bullge | russo laika | spring spaniel |
| beddlington | chihauhua | german short haired | jackerpoo | oldebostonbullge | russoeuropeanlaika | springador |
| bedilington | chihhuahua | german short hr | jackhuahua | oldeenglishbull | russolaika | springadore |
| bedlington | chihouha | german shorthair | jackihuahua | oldeenglishbullge | saarloos | springaler |
| beijon | chihua | german shorthaired | jackpoo | oldenglish | saarloos inuit | springer |
| bekarwal sheep kashmir | chihua hua | german spitz | jackr | oldenglishbull | saarloos wolf | springerdor |
| bekarwalsheepkashmir | chihuaha | german spitz giant | jackrus | oldenglishsheep | saarloos wolfhound | springerdore |
| belg shep | chihuahaua | german spitz klein | jackruseel | oldenglishtymebullgie | saarloosinuit | springerpoo |
| belg shp | chihuahia | german spitz medium | jackrusell | oldetymebullge | saarlooswolf | springspaniel |
| belgain malinois | chihuahu | german spitz mittel | jackruss | oldetymeenglish | saarlooswolfhond | sprockador |
| belgainmalinois | chihuahua | german whaired point | jackrussel | oldevictorianbullge | saarlooswolfhound | sprocker |
| belge griffon | chihuaua | german wire hr | jackrussell | oldspanishbull | saffordshire bull | sprocket |
| belgegriffon | chihuauah | german wired | jackrusser | oldthymebull | saffordshire bull terreir | sproker |
| belgiam malinour | chihuauha | german wired haired | jackrusserl | oldtimebull | saffordshirebull | sprollie |
| belgiammalinour | chihuawa | german wiredhaired | jackrussle | oldtimedorsetbull | saffordshirebullterreir | sprolly |
| belgian griffon | chihuhaha | german wirehaired | jackrusssell | oldtimeenglishbull | sage koochee | sproodle |
| belgian malinois | chihuhahua | german wolfspitz | jackrussuel | oldtimefarmshepherd | sagekoochee | sprspaniel |
| belgian matin | chihuhua | germandeutsche | jackrussuell | oldtyme | sahara | sprug |
| belgian shepard | chihuhuia | germanhunt | jackuahua | oldtyme dorset bull | saint bernard | sringer |
| belgian shepherd | chihuwawa | germanhunter | jackuaua | oldtymebull | saint germain | srpski gonic |
| belgiangriffon | chijhuahua | germanhunting | jackuhaha | oldtymebullgie | saint miguel cattle | srpski trobojni gonic |
| belgianmalinois | chikie | germanlonghaired | jacvk russell | oldtymedorsetbull | saintbernard | srpskigonic |
| belgianmatin | chines crested | germanpinscher | jacvkrussell | oldtynebull | saintgermain | srpskitrobojnigonic |
| belgianshepard | chinescrested | germanroughhaired | jahi | oldvictorianbull | saintmiguelcattle | sspca |
| belgianshepherd | chinese chongqing | germanroughhairedpointing | jahiht | original english bullge | saintongeois | st bernard |
| belgium malinois | chinese crested | germansheep | jamthund | originalenglishbullge | sakhalin | st bernerd |
| belgium shep | chinese crested powderpuff | germansheeperd | japanese chin | ormskirk | saliki | st germain compiegne |
| belgium shepherd | chinese crested puff | germanshep | japanese crest | ormskirk heeler | salki | st germain pointing |
| belgiummalinois | chinese hairless | germanshepard | japanese inu | ormskirkheeler | salucki | staaffy |
| belgiumshep | chinese imperial | germanshepeard | japanese spitz | orsett bull | saluki | staaie |
| belgiumshepherd | chinese powder puff | germansheperd | japanese tosa | orsettbull | salukki | stabbyhoun |
| belgrade | chinesechongqing | germanshephard | japanesechin | orvcharka | samador | stabijhoun |
| belgshep | chinesecrested | germanshepheard | japanesecrest | ostrodlaki gonic barak | samoyad | stabyhoun |
| belgshp | chinesecrestedpowderpuff | germanshepher | japaneseinu | ostrodlakigonicbarak | samoyd | stabyhound |
| belguim shephard | chinesecrestedpuff | germanshepherd | japanesespitz | otter hound | samoyed | staffi |
| belguim shepherd | chinesehairless | germansheppard | japanesetosa | otterhoun | samoyed laphund | staffie |
| belguimshephard | chineseimperial | germanshepperd | japanise spitz | otterhound | samoyedlaphund | staffordhsire |
| belguimshepherd | chinesepowderpuff | germanshepphard | japanisespitz | ovcharka | san lh | staffordhsire bull |
| belguin shepherd | chinua | germansherpard | japenese spits | ovcharka malinois | sanlh | staffordhsirebull |
| belguinshepherd | chinuahua | germansherperd | japenese spitz | ovcharkamalinois | sanshu | staffordshie bull |
| belington | chinuhua | germansherpherd | japenesespits | ovtcharka | sant bernard | staffordshiebull |
| bengal shephard | chion | germanshhephard | japenesespitz | ovtcharka caucasian | santbernard | staffordshire |
| bengalshephard | chipoo | germanshorthair | japinese spitz | ovtcharka central asian | sapniel | staffordshire american |
| bergamasco | chippiparai | germanshorthaired | japinesespitz | ovtcharkacaucasian | sar pei | staffordshire b t |
| bergamese | chiu | germanshorthr | jaxk russell | ovtcharkacentralasian | sarloos | staffordshire blue |
| berger blanc swiss | chiua | germanspitz | jaxkrussell | owczarek mountain | sarpei | staffordshire bt |
| berger de beauce | chiuaha | germanspitzgiant | jck russel | owczarek podhalanski | sarplaninac | staffordshire bul |
| berger de picard | chiuahaua | germanspitzklein | jck russle | owczarekmountain | sarplaninacyugoslavianshepherd | staffordshire bull |
| berger des picard | chiuahua | germanspitzmedium | jckrus | owczarekpodhalanski | sausage dog | staffordshire bulll |
| berger des pyrenees | chiuaua | germanspitzmittel | jckrussel | padenco | sausagedog | staffordshireamerican |
| berger du languedoc | chiuawa | germanwhairedpoint | jckrussle | padengo | scandinavian hound | staffordshireblue |
| berger picard | chiuchia | germanwired | jindo | padenko | scandinavianhound | staffordshirebt |
| berger shetland | chiuhaha | germanwiredhaired | jorkie | papillion | scavenge | staffordshirebul |
| bergerblancswiss | chiuhahua | germanwirehaired | jorky | papillon | scent | staffordshirebull |
| bergerdebeauce | chiuhaua | germanwirehr | jrt | papilon | scent hound | staffordshirebulll |
| bergerdepicard | chiuhua | germanwolfspitz | jrussell | papion | scenthound | stafforshire |
| bergerdespicard | chiuhuahua | germshep | jrussellborder | pappilion | schanuzer | stafforshirebull |
| bergerdespyrenees | chiuhuhua | gey hound | jura hound | pappion | schapendoes | staffs bull |
| bergerdulanguedoc | chiuwawa | geyhound | jurahound | pariah | schauzer | staffsbull |
| bergerpicard | chiwawa | giant schnauzer | kai ken | paron | schi tzu | staffy |
| bergershetland | chiweenie | giant shnauzer | kaiken | parson | schichon | stafy |
| bergr blanc swiss | chiwhawha | giant snauz | kain corso | parson russel | schih tzu | staghound |
| bergrblancswiss | chiwowow | giantschnauzer | kaincorso | parson russell | schihtzu | stb bull |
| berligton | chonzer | giantshnauzer | kairn | parson russell terri | schiller hound | stbbull |
| bermese | chorkie | giantsnauz | kamaskan | parsonrussel | schillerhound | stbernard |
| bernese | chornyi | giardia | kanat | parsonrussell | schillerstovare | stbernerd |
| bernese hound | chow chow | girffon | kangal | parsonrussellterri | schion | stbt |
| bernese mountain | chowaw | glattharidge | kanni | parsons | schipperke | stgermaincompiegne |
| bernesehound | chowchow | gld ret | karabas | parsons j r | schitzu | stgermainpointing |
| bernesemountain | chowie | glden doddle | karabash | parsons russel | schnack | stichelhaar |
| bichalier | chuahaha | glden ret | karabash sheep | parsons russell | schnapen | stoodle |
| bichion | chuahua | glden retriever | karabashsheep | parsonsjr | schnau | stray |
| bichion frise | chug | glden retriver | karabsh | parsonsrussel | schnauser | streeties |
| bichion friz | chuggle | gldendoddle | karaka chan | parsonsrussell | schnautzer | strellufstover |
| bichionfrise | chuhuaha | gldenret | karakachan | parvo | schnauzapoo | stringer spanile |
| bichionfriz | chuhuahua | gldenretriever | karelian bear | parvovirus | schnauzer | stringerspanile |
| bichon | chuhun | gldenretriver | karelianbear | pashmi hound | schnauzerr | stroodle |
| bichon freese | chuiaha | gldret | karst shepherd | pashmihound | schnauzzer | styrian hound |
| bichon freis | chuihuahua | glen imaal | karstshepherd | pastore italiano | schnoodle | styrianhound |
| bichon fries | chuwawa | glen of imaal | kavison | pastoreitaliano | schnoozle | suchon |
| bichon friese | cihuahua | glenimaal | kayleo | pastorel | schnorkie | suliki |
| bichon fris | cimarron | glenofimaal | kc spaniel | patadale | schnuazer | suluki |
| bichon frise | cimarron uruguayo | glepoo | kccs | patedale | schnuzer | suth african bau bau |
| bichon frisee | cimarronuruguayo | gnarl | kcspaniel | paterdale | schon | suthafricanbaubau |
| bichonfreese | cimeco dell etna | gold retreiver | keeshond | pattedale | schoodle | swedish cattle |
| bichonfreis | cimecodelletna | gold retrive | keeshound | patter jack | schweizer laufhun | swedish dachsbracke |
| bichonfries | cinese crested | golden doogle | kelpi | patterdale | schweizerlaufhun | swedish elkhound |
| bichonfriese | cinesecrested | golden irish | kelpie | patterdalt | scischon | swedish fohound |
| bichonfris | cireco dell etna | golden ret | kelpy | patterjack | scnauzer | swedish lapphund |
| bichonfrise | cirecodelletna | golden reteiver | kemmer | patterpoo | scnhauzer | swedish lapphund imp |
| bichonfrisee | cirneco | golden retevier | kennel | paw | scnoodle | swedish vallhund |
| bichonpoo | cirneco del etna | golden retreaver | kerry blue | peckeniese | scoodle | swedish vul hound |
| bichpoo | cirneco dell etna | golden retreiver | kerry hunting hound | pedigr | scot bull | swedishcattle |
| bicoh | cirneco delletna | golden retrieve | kerryblue | pedigree | scotbull | swedishdachsbracke |
| bicsion | cirnecodeletna | golden retriever | kerryhuntinghound | peekapoo | scott bull | swedishelkhound |
| biewer | cirnecodell etna | golden retriver | king chales spaniel | pekazu | scottbull | swedishfohound |
| bijion friese | cirnecodelletna | goldendoogle | king charles | peke | scottie | swedishlapphund |
| bijionfriese | claw | goldendoor | king charles cav | pekenise | scottish border | swedishlapphundimp |
| bijon | clumber | goldenirish | king charles cavaleir | pekepoo | scottish cairn | swedishvallhund |
| biladi | coarse hair | goldenpoo | king charles cavaler | pekinees | scottish carin | swedishvulhound |
| billy | coarsehair | goldenret | king charles cavalier | pekinese | scottish hound | swiis shepherd |
| binscher | coca poo | goldenreteiver | king charles cavalier spaniel | pekinesse | scottish rough | swiisshepherd |
| biochen | cocapoo | goldenretevier | king charles cavelier | pekingese | scottish smooth | swiss hound |
| bischon | cockachon | goldenretreaver | king charles caverle | pekingnese | scottish terier | swiss laufhund jura |
| bischon frise | cockador | goldenretreiver | king charles caverler | pembroke | scottishborder | swiss laufhunds |
| bischonfrise | cockalier | goldenretrieve | king charles caviller | pembrokeshire | scottishcairn | swiss mountain |
| bischonpoo | cockapoo | goldenretriever | king charles spaniel | pembrokshire | scottishcarin | swiss shepherd |
| biscon | cockeerpoo | goldenretriver | king corso | pembrooke | scottishhound | swiss sherpard |
| biscon frise | cockepoo | golderdors | king shepherd | perdigueiro portuguese | scottishrough | swiss short hair |
| bisconfrise | cocker | goldern retriever | kingchalesspaniel | perdigueiroportuguese | scottishsmooth | swiss short haired pinscher |
| bishion | cockpoo | goldernretriever | kingcharles | perdiguero de burgos | scottishterier | swiss white shepherd |
| bishon | cokapoo | goldretreiver | kingcharlescav | perdiguero navarro | scotty | swisshound |
| bishon freesh | coker | goldretrive | kingcharlescavaleir | perdiguerodeburgos | sealydale | swisslaufhundjura |
| bishon frise | colie | good boy | kingcharlescavaler | perdigueronavarro | sealyham | swisslaufhunds |
| bishonfreesh | coliie | good girl | kingcharlescavalier | perduco | segugio hound | swissmountain |
| bishonfrise | collar | goodboy | kingcharlescavalierspaniel | perro cimarron | segugio italiano | swissshepherd |
| bitch | colley | goodgirl | kingcharlescavelier | perro de paster | segugio swiss mountian | swisssherpard |
| bitchon | colli | gorden setter | kingcharlescaverle | perro de pastor | segugiohound | swissshorthair |
| bite | collie | gordensetter | kingcharlescaverler | perro de presa | segugioitaliano | swissshorthairedpinscher |
| bivhon | collir | gordon setter | kingcharlescaviller | perro de presa canario | segugios italianos | swisswhiteshepherd |
| bizanian | colllie | gordonsetter | kingcharlesspaniel | perro de presa mallorquin | segugiositalianos | syberian |
| black and tan | colloe | gos d atura catalan | kingcorso | perro ratonero andaluz | segugioswissmountian | sydney silky |
| black fell | colly | gosdaturacatalan | kingshepherd | perrocimarron | seltie | sydneysilky |
| black forest hound | combai | gran mastin de borinquen | kirhiz | perrodepaster | serbian hound | tackel |
| black russian | coockerpoo | grand basset griffon | kishu | perrodepastor | serbian sheep | tahltan bear |
| black tan | coodle | grand basset griffon vendeen | kishu ken | perrodepresa | serbian tricolour hound | tahltanbear |
| blackandtan | cookapoo | grand bassett | kishuken | perrodepresacanario | serbianhound | taigan |
| blackfell | cookerpoo | grand bassett griffon vendeen | klee kai | perrodepresamallorquin | serbiansheep | tail |
| blackforesthound | coolie | grand bleu de gascogne | klee kia | perroratoneroandaluz | serbiantricolourhound | tamaskan |
| blackmouth cur | coon hound | grand bleu de gascoigne | kleekai | persian sighthound | serra de aires mountain | tape worm |
| blackmouthcur | coonhound | grand bleu gascogne | kleekia | persiansighthound | serradeairesmountain | tapeworm |
| blackrussian | copoochon | grand bleus de gascogne | klein german spitz | peruvian hairless | setland sheep | tatra mountain |
| blacktan | corg | grand griffon vendeen | kleingermanspitz | peruvian inca orchid | setlandsheep | tatra shepherd |
| blanc swiss | corgi | grandbassetgriffon | kng charles | peruvianhairless | setter | tatra shiper |
| blancswiss | corgie | grandbassetgriffonvendeen | kngal | peruvianincaorchid | sha pei | tatramountain |
| bledington | corgy | grandbassett | kngcharles | petit basset griffon | shai pei | tatrashepherd |
| bleu de gascogne | cormerlyn | grandbassettgriffonvendeen | koiinderhundge | petit basset griffon vendeen | shaipei | tatrashiper |
| bleu gascogne | corso italiano | grandbleudegascogne | kokoni | petit bleu de gascogne | shapei | tchiorny |
| bleudegascogne | corsoitaliano | grandbleudegascoigne | kokonix | petit bleus de gascogne | shapi | teagle |
| bleugascogne | coton de telaur | grandbleugascogne | komondor | petit brabancon | shar pai | tebetan |
| bleus de gascogne | coton de tulear | grandbleusdegascogne | kooikehondje | petit gascon | shar pei | teckel |
| bleusdegascogne | coton du tulear | grande basset griffon vendeen | kooiker | petit gascon saintongeois | shar peis | tenterfield |
| blood bull | coton noodle | grandebassetgriffonvendeen | kooikerhondje | petit griffon | shar pey | teriuvum shepard |
| blood hound | cotondetelaur | grandgriffon bassetvendeen | kooikerhonege | petit griffon bleu de gascogne | shari pei | teriuvumshepard |
| bloodbull | cotondetulear | grandgriffonbassetvendeen | kookier hondje | petitbassetgriffon | sharipei | terreir |
| bloodhound | cotondutulear | grandgriffonvendeen | kookierhondje | petitbassetgriffonvendeen | sharpai | terrier |
| blue alpama | cotonnoodle | granmastindeborinquen | koolie | petitbleudegascogne | sharpay | terrior |
| blue belton | cotonoodle | grayhound | korean jindo | petitbleusdegascogne | sharpe | terrir |
| blue gascon | cotton de tulear | great anglo french hound | koreanjindo | petitbrabancon | sharpee | tervueren |
| blue heeler | cottondetulear | great anglo french tricolour hound | korthal griffon | petite basset griffon | sharpei | tervuren |
| blue kerry | courser | great anglo french white and black hound | korthalgriffon | petite brabancon griffon | sharpeis | thai bangkaew |
| blue lacey | coursing hound | great anglo french white and orange hound | korthall griffon | petite griffon basset hound | sharpey | thai bankaew |
| blue lacy | coursinghound | great dain | korthallgriffon | petitebassetgriffon | sharpi | thai ridgeback |
| blue maril | cream spitz | great dane | korthals | petitebrabancongriffon | sharpie | thaibangkaew |
| blue merle | creamspitz | great pyrenees | korthals griffen | petitegriffonbassethound | sharplaninec | thaibankaew |
| blue picardy | cretan hound | great pyrenese | korthals griffin | petitgascon | shcnauzer | thairidgeback |
| blue roan spaniel | cretanhound | great swiss cattle | korthals griffon | petitgasconsaintongeois | sheba enu | tiara teddy bear |
| bluealpama | croatian sheep | great swiss mountain | korthals pointing griffon | petitgriffon | sheba inu | tiara teddybear |
| bluebelton | croatian shepherd | great swss mountn | korthalsgriffen | petitgriffonbleudegascogne | shebaenu | tiarateddybear |
| bluegascon | croatiansheep | greatanglofrenchhound | korthalsgriffin | petterdae | shebainu | tibertanter |
| blueheeler | croatianshepherd | greatanglofrenchtricolourhound | korthalsgriffon | phalene | shebauno | tibetan kyiapso |
| bluekerry | cspaniel | greatanglofrenchwhiteandblackhound | korthalspointinggriffon | pharaoh hound | sheeba inu | tibetan spaniel |
| bluelacey | cuckerpoo | greatanglofrenchwhiteandorangehound | kosovan mountain | pharaohhound | sheebainu | tibetan ter |
| bluelacy | curly coat | greatdain | kosovanmountain | pharoah hound | sheep australian | tibetan terier |
| bluemaril | curly coated retreiver | greatdane | krasky ovcar | pharoahhound | sheep belgian | tibetankyiapso |
| bluemerle | curly coated retriever | greater swiss mountain | kraskyovcar | pharoh hound | sheep buhund | tibetanspaniel |
| bluepicardy | curly coated retriver | greaterswissmountain | kromfohrlande | pharohhound | sheep bulgarian | tibetanter |
| blueroanspaniel | curlycoat | greatpyrenees | kromfohrlander | picanese | sheep carpathian | tibetanterier |
| bodeguero | curlycoatedretreiver | greatpyrenese | kromfohrländer | picardy | sheep catalan | tibetian spaniel |
| bodenco | curlycoatedretriever | greatswisscattle | kuvasz | picardy sheep | sheep caucasian | tibetianspaniel |
| boer boel | curlycoatedretriver | greatswissmountain | kyi apso | picardy shepherd | sheep central asian | tick |
| boerbel | cvalier springer | greatswssmountn | kyi leo | picardy spaniel | sheep croatian | timber wolf |
| boerbeol | cvalierspringer | greek domestic | kyiapso | picardysheep | sheep dag | timberwolf |
| boerboel | cvlier king charles | greek hair hound | kyileo | picardyshepherd | sheep dutch | toller |
| boerboul | cvlierkingcharles | greek hairhound | lab ret | picardyspaniel | sheep greek | tolling retriever |
| boerbul | czech wolf | greek hare hound | lab retreiver | piccolo levriero italiano | sheep hungarian | tollingretriever |
| bohemian pointing griffon | czechoslovakian wolf | greek harehound | lab retriever | piccololevrieroitaliano | sheep karakachan | tornjak |
| bohemian shepherd | czechoslovakianwolf | greek hound | lab retriver | pikenese | sheep komondor | tosa |
| bohemianpointinggriffon | czechwolf | greek kokoni | labador | pincer | sheep maremma | tosa inu |
| bohemianshepherd | d de bordeaux | greek sheep | labarador | pincher | sheep new zealand huntaway | tosainu |
| bolanese | da serra da estrela | greek shepherd | labarador lurcher | pinsc | sheep norwegian | tracker |
| bolognaisie | da serra de aires | greekdomestic | labaradorlurcher | pinsch | sheep old english | trail hound |
| bologne | dach | greekhairhound | labardor | pinscher | sheep polish | trailhound |
| bolognese | dachaund | greekharehound | labora | pinscher austrian | sheep polish lowland | transylvanian hound |
| bologneseac | dachchund | greekhound | labra | pinscher austrian short haired | sheep romanian | transylvanian scent hound |
| boloka | dachhund | greekkokoni | labradinger | pinscher carlin | sheep romanian bukovina | transylvanianhound |
| bolonka | dachipoo | greeksheep | labradire | pinscher german | sheep romanian mioritic | transylvanianscenthound |
| bone | dachs | greekshepherd | labradoddle | pinscheraustrian | sheep shetland | treat |
| borador | dachsand | greenland esquimau | labradooddle | pinscheraustrianshorthaired | sheep welsh | tree walker coonhound |
| bordeaubull | dachsbracke | greenland hound | labradoor | pinschercarlin | sheepaustralian | treeing tennessee |
| bordegerro andaluz | dachschund | greenlandesquimau | labrador | pinschergerman | sheepbelgian | treeing walker coonhound |
| bordegerroandaluz | dachshaund | greenlandhound | labrador retreiver | pinser | sheepbuhund | treeingtennessee |
| border collie | dachshound | gretriever | labrador retrevier | pinsher | sheepbulgarian | treeingwalkercoonhound |
| bordercollie | dachshund | grey hound | labrador retriever | pinzer | sheepcarpathian | treewalkercoonhound |
| bordoodle | dachsun | greyhoud | labrador type | pit bull | sheepcatalan | tricolour |
| borkie | dachsund | greyhound | labradore | pit staff | sheepcaucasian | trigg hound |
| borzoi | dachsy | griffin | labradorret | pitbull | sheepcentralasian | trigghound |
| bosanski | dachund | griffon | labradorretreiver | pitbull bull | sheepcroatian | tsvetnaya bolonka |
| boston bull | dacshund | griffon basset vendeen grand | labradorretrevier | pitbullbull | sheepdag | tsvetnayabolonka |
| boston terr | dakotah shepherd | griffon basset vendeen petit | labradorretriever | pitstaff | sheepdutch | tulear |
| bostonbull | dakotahshepherd | griffon basset vendeen unspecified | labradortype | pitt | sheepgreek | turkish kangal |
| bostonterr | dalamatian | griffon belge | labras | pitt bull | sheephungarian | turkish sheep |
| bourbonnais | dalmatian | griffon belgian griffon belge | labrashepherd | pittball | sheepkarakachan | turkish shep |
| bourdeubull | dalmation | griffon bleu de gascogne | labrdaor | pittbull | sheepkomondor | turkishkangal |
| bouvier | dandie dinmont | griffon bleu de gascogne grand | labret | plott hound | sheepmaremma | turkishsheep |
| bouvier de flandres | dandiedinmont | griffon bleu de gascogne petit | labretr | plotthound | sheepnewzealandhuntaway | turkishshep |
| bouvier des ardennes | dane | griffon bleu de gascoigne | labretreiver | plumber terrrier | sheepnorwegian | tyrolean hound |
| bouvier des flandres | danish swedish farm | griffon brabancon | labretriever | plumberterrrier | sheepoldenglish | tyroleanhound |
| bouvier suisse | danishswedishfarm | griffon brussels | labretriver | plummer | sheepoo | tyroler |
| bouvierdeardennes | daschaund | griffon bruxblliouf | labrit | pncher | sheeppolish | tyroler bracke |
| bouvierdeflandres | daschhound | griffon bruxellois | labro | pochan | sheeppolishlowland | tyrolerbracke |
| bouvierdesardennes | daschound | griffon de vendeen | labroa | poddle | sheepromanian | utonagan |
| bouvierdesflandres | daschund | griffon fauve de bretagne | labroador | podenca | sheepromanianbukovina | utonagon |
| bouviersuisse | daserradaestrela | griffon nivernais | labrodor | podenco | sheepromanianmioritic | valee sheep |
| bow wow | daserradeaires | griffon pointing | labrrador | podenco andaluz | sheepshetland | valeesheep |
| bowwow | dashaund | griffon rough haired | ladra | podenco bull | sheepwelsh | valley bull |
| boxador | dashhound | griffon vende | laeken | podenco canario | sheiba inu | valleybull |
| boxer | dashound | griffon vendeen | laekenois | podenco ibicenco | sheibainu | varkhond |
| boxodor | dashund | griffon vendeen grand | lagatto romagnolo | podenco labrador | shelter | vendeen basset |
| boykin | datch hound | griffon vendeen petit | lagattoromagnolo | podenco possibly | shelti | vendeenbasset |
| bracco | datchhound | griffon wire hr pointing | lagoto | podencoandaluz | sheltie | vendn grnd |
| bracco italia | dauchand | griffonbassetvendeengrand | lagotto | podencobull | shelty | vendn peti |
| braccoitalia | dauchound | griffonbassetvendeenpetit | lagotto romagholo | podencocanario | shepa dor | vendngrnd |
| bracke | dauchund | griffonbassetvendeenunspecified | lagotto romagnolo | podencoibicenco | shepador | vendnpeti |
| braid | dausand | griffonbelge | lagottoromagholo | podencolabrador | shepard | vet |
| braque belge | dauschaudnd | griffonbelgiangriffonbelge | lagottoromagnolo | podencopossibly | sheperd | victoria bull |
| braque d auvergne | dauschund | griffonbleudegascogne | lahasa apso | podengo | sheperkita | victoriabull |
| braque de auvergne | daushand | griffonbleudegascognegrand | lahasaapso | podengo canario | sheph | victorian bull |
| braque du bourbonnais | daushound | griffonbleudegascognepetit | laika | podengo pequeno | shephard | victorianbull |
| braque dupuy | dautchund | griffonbleudegascoigne | lakeland | podengocanario | shepherd | villano de las encartaciones |
| braque francais | dawg | griffonbrabancon | lakenois | podengopequeno | shepherd american white | villanodelasencartaciones |
| braque francais de grand taille | dax | griffonbrussels | lakeview bullge | podhalanski mountain | shepherd anatolian | visla |
| braque francais de petite taille | daxi | griffonbruxblliouf | lakeviewbullge | podhalanskimountain | shepherd australian | visler |
| braque saint germain | daxon | griffonbruxellois | lamalese | poimenikos | shepherd beauce bas rouge | viszla |
| braquebelge | daxy | griffondevendeen | lancashire heal | pointer | shepherd belgian | vizla |
| braquedauvergne | ddbordeaux | griffonfauvedebretagne | lancashire healer | pointing gascogne | shepherd belgian groenendael | vizladore |
| braquedeauvergne | ddebordeaux | griffonnivernais | lancashire heeler | pointing griffon | shepherd belgian laekenois | vizler |
| braquedubourbonnais | de ardennes | griffonpointing | lancashireheal | pointing pyrenean | shepherd belgian malinois | vizsla |
| braquedupuy | de bordeaux | griffonroughhaired | lancashirehealer | pointing w haired griffon | shepherd belgian tervuren | vizslador |
| braquefrancais | de bordeoux | griffonvende | lancashireheeler | pointinggascogne | shepherd bergamasco | volpino |
| braquefrancaisdegrandtaille | de bordeux | griffonvendeen | lancs heeler | pointinggriffon | shepherd caucasian | volpino italiano |
| braquefrancaisdepetitetaille | de castro laboreiro | griffonvendeengrand | lancsheeler | pointingpyrenean | shepherd central asian | volpinoitaliano |
| braquesaintgermain | de pont audemer | griffonvendeenpetit | landceer | pointingwhairedgriffon | shepherd dutch | vorstehhund |
| brazilian bulge | de presa canario | griffonwirehrpointing | landseer | poitevin | shepherd east european | walk |
| brazilian molosser | deardennes | griffoodle | landseer newfoundland | poland staff | shepherd english | walkies |
| brazilian pressa | debordeaux | grifon | landseernewfoundland | polandstaff | shepherd german | warren hound |
| brazilianbulge | debordeoux | grman shepard | landser newfoundland | polish chart polski | shepherd italian | warren podengo |
| brazilianmolosser | debordeux | grman shepered | landsernewfoundland | polish hound | shepherd karst | warrenhound |
| brazilianpressa | debordo | grmanshepard | lapal | polish hunting | shepherd king | warrenpodengo |
| breaded collie | decastrolaboreiro | grmanshepered | lapinporokoira | polish lland sheepdg | shepherd malinois | water american |
| breadedcollie | deer hound | groendal sherpherd | lapland rein | polish lowland sheep | shepherd old time farm | water friesian |
| breaton | deerhound | groendalsherpherd | lapland reindeer | polish mountain | shepherd shiloh | water hungarian puli |
| bress | depontaudemer | groenendael | laplandrein | polish sheep | shepherd siberian | water irish |
| breton | depresacanario | groodle | laplandreindeer | polish shepherd | shepherd swiss white | water portuguese |
| brettan | deu bordaux | grooming | lapphund | polish tatra sheep | shepherd yugoslavian | water spaniel |
| bretton | deubordaux | grosser munsterlander | lapponian herder | polishchartpolski | shepherdamericanwhite | water spanish |
| briard | deuchand | grossermunsterlander | lapponianherder | polishhound | shepherdanatolian | wateramerican |
| briewer | deutsch langhaar | growl | laps tzu | polishhunting | shepherdaustralian | waterfriesian |
| brindle | deutsche | grundale | lapso | polishllandsheepdg | shepherdbeaucebasrouge | waterhungarianpuli |
| briquet | deutsche bracke | grunlandshund | lapstzu | polishlowlandsheep | shepherdbelgian | waterirish |
| briquet griffon vendeen | deutschebracke | gsd | larbrado | polishmountain | shepherdbelgiangroenendael | waterportuguese |
| briquetgriffonvendeen | deutscher | gshd | largemunsterlander | polishsheep | shepherdbelgianlaekenois | waterspaniel |
| britany | deutschlanghaar | guard serra da estrela | lasa apso | polishshepherd | shepherdbelgianmalinois | waterspanish |
| britin spaniel | dingo | guardserradaestrela | lasaapso | polishtatrasheep | shepherdbelgiantervuren | wauzer |
| britinspaniel | doberman | hairless chinese crested | lasha | polski owczarek | shepherdbergamasco | weaton |
| british bull | dobermann | hairless puff | laso apso | polskiowczarek | shepherdcaucasian | weimanarer |
| britishbull | dobi | hairlesschinesecrested | lasoapso | pom | shepherdcentralasian | weimaraner |
| britney spaniel | dobie | hairlesspuff | lasopoo | pomapoo | shepherddutch | weimarar |
| britneyspaniel | dog | halden hound | lassapso | pomaramian | shepherdeasteuropean | weimararner |
| brittanny | dogudbordeaux | haldenhound | lasso apso | pomaranian | shepherdenglish | weimariner |
| brittany | dogue brasileiro | haldenstovare | lassoapso | pomchee | shepherdgerman | weimer |
| brittany american | dogue de boardeux | hamilton hound | latvian hound | pomchi | shepherditalian | weimeramer |
| brittany french | dogue de bordeau | hamilton stovare | latvianhound | pomchy | shepherdkarst | weimeraner |
| brittanyamerican | dogue de bordeaux | hamilton strovare | layland | pomeagle | shepherdking | weinemarrer |
| brittanyfrench | dogue de bordeoux | hamiltonhound | lbradour | pomer | shepherdmalinois | weinmarer |
| brohol | dogue de bordeux | hamiltonstovare | lbrodoole | pomeraian | shepherdoldtimefarm | weirmeraner |
| broholmer | dogue debordeau | hamiltonstrovare | learcher | pomeranain | shepherdshiloh | welsh cardigan |
| brolley | dogue debordeaux | hanovarian hound | legotta | pomeranian | shepherdsiberian | welsh springer |
| bruno de jura | dogue do bordeaux | hanovarian mountain hound | leonberger | pomeranion | shepherdswisswhite | welsh sprngr |
| brunodejura | doguebrasileiro | hanovarianhound | leonburger | pomeranium | shepherdyugoslavian | welshcardigan |
| brussels griffon | doguedeboardeux | hanovarianmountainhound | leopard cur | pomeraniun | shepie | welshpembroke |
| brusselsgriffon | doguedebordeau | hanover hound | leopardcur | pomerian | shepoo | welshsheep |
| bruxelles griffon | doguedebordeaux | hanoverhound | lercher | pomerianan | sheppard | welshspringer |
| bruxellesgriffon | doguedebordeoux | hanoverian hound | levesque | pomernian | shetland sheep | welshsprngr |
| bruxellois | doguedebordeux | hanoverian scent hound | lewellen setter | pomerpoo | shetlandsheep | west h land |
| bruxellois griffon | doguedobordeaux | hanoverian scenthound | lewellensetter | pommeranian | shhitzu | west high land |
| bruxelloisgriffon | doodle | hanoverianhound | lhahso apso | pomopoo | shi chon | west highland |
| bscon | dorgi | hanoverianscenthound | lhahsoapso | pompoo | shi poo | west highland terr |
| bshon frise | dorgie | harehound | lhaired point | pompsky | shi tsu | west highland white |
| bsset | dorset bull | harlequin hound | lhairedpoint | pomshi | shi tzu | west higland white |
| bston terror | dorset old english bull | harlequin pinscher | lhapchon | pomski | shi zhu | west scottish highland |
| bucovina | dorset old t bull | harlequinhound | lhapso | pomsky | shi zu | west siberian laika |
| bugg | dorset old tyme bull | harlequinpinscher | lhasa | ponchie | shia tsu | weste |
| buhund | dorset old tyme bullge | harness | lhasa apaso | pont audemer | shiatsu | western terrie |
| bukovina | dorset olde tyme bullge | harrier | lhasa aposo | pontaudemer | shib inu | westernterrie |
| bulgarian shepard | dorsetbull | harrier hound | lhasa aps | poo | shiba | westhighland |
| bulgarian shepherd | dorsetoldenglishbull | harrierhound | lhasa apsa | poo chon | shiba ibu | westhighland white |
| bulgarianshepard | dorsetoldetymebullge | havamalt | lhasa apso | pooch | shiba imu | westhighlandterr |
| bulgarianshepherd | dorsetoldtbull | havana silk | lhasa apsu | poochi | shiba inn | westhighlandwhite |
| bull american | dorsetoldtymebull | havanais | lhasaapaso | poochie | shiba inu | westhiglandwhite |
| bull arab | dorsetoldtymebullge | havanasilk | lhasaaposo | poochion | shiba inuit | westhland |
| bull australian | douge de bordeaux | havanese | lhasaaps | poochon | shiba uno | westi |
| bull french | douge de boudeuridgeback | havanoodle | lhasaapsa | poochon frise | shibaibu | westichon |
| bull german shepherd | dougedebordeaux | havaton | lhasaapso | poochonfrise | shibaimu | westie |
| bull italian | dougedeboudeuridgeback | havenese | lhasaapsu | poodle | shibainn | westipoo |
| bull mallorquin | doughue de bordaux | havense | lhasapoo | poogle | shibainu | westphalian |
| bull mestiff | doughuedebordaux | hawaiian poi | lhasha apso | pooschon | shibainuit | westphalian dachsbracke |
| bull miniatr | dougue de bordeaux | hawaiianpoi | lhashaapso | pooshi | shibauno | westphaliandachsbracke |
| bull old time | douguedebordeaux | heartworm | lhaso | pooshion | shibi inu | westscottishhighland |
| bull old tyme | doxiepoo | heeler | lhaso apso | pooshon | shibiinu | westsiberianlaika |
| bull spanish | drentsche partridge | heeler blue | lhasoapso | pootalian | shibinu | westy |
| bull terrie | drentschepartridge | heelerblue | lhasoliar | poperanian | shibu inu | westypoo |
| bull terrrier | drentse | hellenic hound | lhasso apso | porcelaine | shibuinu | wetterhoun |
| bull victorian | drentse partridge | hellenichound | lhassoapso | portegese water | shichi | wetterhound |
| bullador | drentse patrijshond | hellenikos | lithuanian hound | portegesewater | shichon | whaeten |
| bullamerican | drentsepartridge | herder | lithuanianhound | portugese podengo | shih hua hua | wheatan |
| bullarab | drentsepatrijshond | himalayan sheep | little lion | portugese sheep | shih poo | wheaten |
| bullaustralian | drever | himalayansheep | littlelion | portugese water | shih schon | wheatern |
| bulldpg | du bordeaux | hokkaido | llasa apso | portugesepodengo | shih stzu | wheaton |
| bullfrench | dubordeaux | holland shepherd | llasaapso | portugesesheep | shih suzi | whechon |
| bullge | duck retriever | hollandshepherd | llewellin | portugesewater | shih szu | wheeton |
| bullgermanshepherd | duck tolling retreiver | hook worm | lncashire heal | portugues podengo | shih tsu | whelp |
| bullitalian | duck tolling retriever | hookworm | lncashireheal | portugues water | shih tszu | whetan |
| bullmallorquin | duckretriever | hound | lng haired | portuguese cattle | shih tzi | whip worm |
| bullmasstiff | ducktoller | hound afghan | lnghaired | portuguese guard | shih tzn | whipet |
| bullmastive | ducktollingretreiver | hound arabian | logotto | portuguese hound | shih tzu | whippet |
| bullmestiff | ducktollingretriever | hound balkan | logotto romagnolo | portuguese hunting | shih zhu | whippett |
| bullminiatr | due brasileiro | hound basset | logottoromagnolo | portuguese imp | shih ztu | whippit |
| bullnese | duebrasileiro | hound bavarian mountain | long coat | portuguese perdigueiro | shih zu | whipworm |
| bullog english | dutch german shepherd | hound bernese | long coated | portuguese podengo | shih zue | white swiss shepherd |
| bullogenglish | dutch herd | hound black forrest | long hair | portuguese podengo pequeno | shihhuahua | whiteshepherd |
| bulloldtime | dutch herder | hound bleu de gascogne grand | long haired | portuguese rabbit | shihpoo | whiteswissshepherd |
| bulloldtyme | dutch malanois | hound canary warren podenco canario | long hr | portuguese sheep | shihschon | whoodle |
| bullspanish | dutch partridge | hound dutch tulip hollandse tulphond markiesje | long smooth | portuguese watch | shihstzu | whwt |
| bullterrie | dutch schapendoes | hound french white and black | longcoat | portuguese water | shihsuzi | wiemaraner |
| bullterrrier | dutch sheep | hound german | longcoated | portuguesecattle | shihszu | wimeraner |
| bullvictorian | dutch shepard | hound greek | longhair | portugueseguard | shiht zhu | wippet |
| bully | dutch sheperd | hound halden | longhaired | portuguesehound | shihtsu | wire coat |
| bully kuttas | dutch shepherd | hound hamilton | longhr | portuguesehunting | shihtszu | wire foterrie |
| bullykuttas | dutch smoushond | hound hanoverian | longsmooth | portugueseimp | shihtzhu | wire haired |
| bulue campeiro | dutch stabyhoun | hound ibizan | lonhair | portugueseperdigueiro | shihtzi | wire hr |
| buluecampeiro | dutch strellufstover | hound istrian | loughlander | portuguesepodengo | shihtzn | wirecoat |
| burgos pointing | dutch water friesian | hound italian | low chen | portuguesepodengopequeno | shihtzu | wired hair |
| burgospointing | dutch waterfowl | hound latvian | lowchen | portugueserabbit | shihzhu | wired haired |
| burmese | dutchgermanshepherd | hound pharaoh | lowchen little lion | portuguesesheep | shihztu | wiredhair |
| burmese montain | dutchherd | hound polish | lowchenlittlelion | portuguesewatch | shihzu | wiredhaired |
| burmese mountain | dutchherder | hound portuguese | lowland polish | portuguesewater | shihzue | wirefo |
| burmese moutain | dutchhound | hound posavina | lowlandpolish | portuguespodengo | shika inu | wirefoterrie |
| burmesemontain | dutchmalanois | hound russian | lrcher | portugueswater | shikainu | wirefox |
| burmesemountain | dutchound | hound russian harlequin | lucher | posavatz hound | shikoku | wirehaired |
| burmesemoutain | dutchpartridge | hound schiller | lundehund | posavatzhound | shin tzu | wirehr |
| burnese mountain | dutchschapendoes | hound serbian | lurcher | posavina hound | shintzu | wirewhaired |
| burnesemountain | dutchsheep | hound slovakian | lurhcer | posavinahound | shipoo | wolf |
| busion | dutchshepard | hound spanish | lurtcher | pot hound | shiranian | wolf british |
| c spaniel | dutchsheperd | hound trail | macedonian mountain | potenco | shischon | wolf czechoslovakian |
| ca de bou | dutchshepherd | hound trigg | macedonianmountain | pothound | shiscon | wolf hound |
| cadebou | dutchsmoushond | hound tyrolean | macellaio sicillian branchiero | pouchon | shishon | wolf inuit |
| cain corso | dutchstabyhoun | houndafghan | macellaiosicillianbranchiero | powder puff | shishonpoo | wolf siberian |
| caincorso | dutchstrellufstover | houndarabian | magyar agar | powderpuff | shishpoo | wolfbritish |
| caine corso | dutchwaterfowl | houndbalkan | magyaragar | prague krysarik | shit tzu | wolfczechoslovakian |
| cainecorso | dutchwaterfriesian | houndbasset | mahogany japenese | prague rat hunter | shit zhu | wolfhound |
| cairn | east european shepherd | houndbavarianmountain | mahoganyjapenese | prague ratter | shit zu | wolfhound czechoslovakian |
| cairne | east siberia laika | houndbernese | mahogony japanese | praguekrysarik | shitizu | wolfhound irish |
| campeiro | east siberian laika | houndblackforrest | mahogonyjapanese | praguerathunter | shitsu | wolfhound russian |
| cana corsa | easteuropeanshepherd | houndbleudegascognegrand | majorca shepherd | pragueratter | shitszhu | wolfhound saarloos |
| cana corso | eastsiberialaika | houndcanarywarrenpodencocanario | majorcashepherd | praque ratter | shittzu | wolfhoundczechoslovakian |
| canaan | eastsiberianlaika | hounddutchtuliphollandsetulphondmarkiesje | mal shi | praqueratter | shitz | wolfhoundirish |
| canachan | eatern europe shepherd | houndfrenchwhiteandblack | malamut | prazsky krysavik | shitz chu | wolfhoundrussian |
| canacorsa | eaterneuropeshepherd | houndgerman | malamute | prazskykrysavik | shitz poo | wolfhoundsaarloos |
| canacorso | ebt | houndgreek | malinois | presa canairo | shitz zu | wolfinuit |
| canadian eskimo | egyptian canaan | houndhalden | malionois | presa canaria | shitz zue | wolfsiberian |
| canadian inuit | egyptian pharoh hunting hound | houndhamilton | mallanoy | presa canario | shitzchu | wolfspitz |
| canadianeskimo | egyptiancanaan | houndhanoverian | mallinios | presa canary | shitzhu | wood green |
| canadianinuit | egyptianpharohhuntinghound | houndibizan | mallinois | presacanairo | shitzpoo | woodgreen |
| canarian warren hound | elkhound | houndistrian | mallinos | presacanaria | shitzsu | woodle |
| canarianwarrenhound | elkhound norwegian | hounditalian | mallorquin bull | presacanario | shitzu | woof |
| canary perro | elkhoundnorwegian | houndlatvian | mallorquin ca de bestiar | presacanary | shitzy | woolf |
| canaryperro | eng bull | houndpharaoh | mallorquinbull | preso canario | shitzzu | wormer |
| cancorse | engbull | houndpolish | mallorquincadebestiar | presocanario | shitzzue | wowzer |
| cane corsa | englidh bull | houndportuguese | malmute | pressa canaria | shiu tzu | wozer |
| cane corso | englidhbull | houndposavina | malnois | pressa canario | shiutzu | wst highland |
| cane cosa | engligh bull | houndrussian | malshi | pressa carndio | shiz | wsthighland |
| cane cosrso | englighbull | houndrussianharlequin | malshie | pressa mallorquin | shiz tzu | xolo |
| cane de pastore | english bul | houndschiller | malshih | pressacanaria | shiz zu | xoloitzcuintle |
| canecorsa | english bull | houndserbian | maltalier | pressacanario | shizhu | xoloitzcuintli |
| canecorso | english bull terr | houndslovakian | maltchu | pressacarndio | shizpo | yap |
| canecosa | english setter | houndspanish | maltease | pressamallorquin | shiztzhu | yarmouth toller |
| canecosrso | english sheep | houndtrail | malteese | presser bull | shiztzu | yarmouthtoller |
| canedepastore | english shepherd | houndtrigg | maltepoo | presser canary | shizu | ykrt |
| caniche | english spaniel | houndtyrolean | maltese | presser coursa | shizzu | yo chon |
| caniche bichon | english springer | hovawart | maltesse | presserbull | shnauzer | yochon |
| canichebichon | english springer spainel | hovervart | maltez | pressercanary | shnoodle | yokie |
| canid | english springer spanial | howl | malti poo | pressercoursa | shnorkie | yokiepoo |
| canine | english staffordshire bull | hrvatski ovcar | maltipoo | preza canario | shorkie | yoodle |
| canis | englishblack tan | hrvatskiovcar | maltise | prezacanario | shorky | yorke |
| canne corso | englishblacktan | hungarian hound | mans best | prinian mountain | short coat | yorki |
| cannecorso | englishbul | hungarian komondor | mansbest | prinianmountain | short hair | yorki poo |
| cano corsa | englishbull | hungarian kuvasz | maremma | prson | short haired | yorkie |
| cano corso | englishbullterr | hungarian mudi | maremma elkhound | ptbull | short haired pointing | yorkie poo |
| canocorsa | englishsetter | hungarian puli | maremma sheep | pudel | short haired segugio | yorkiepoo |
| canocorso | englishsheep | hungarian pulis | maremmaelkhound | pug | short hr | yorkipoo |
| cao de agua | englishshepherd | hungarian pumi | maremmasheep | puli | shortcoat | yorkishire |
| cao de agua portie | englishspaniel | hungarian sheep | maremmo abruzzese | pulik | shorthair | yorkpoo |
| cao de castro laboreiro | englishspringer | hungarian short haired | maremmoabruzzese | pumi | shorthaired | yorkshie |
| caodeagua | englishspringerspainel | hungarian visla | massai cattle | pumi mudi | shorthairedpointing | yorkshire |
| caodeaguaportie | englishspringerspanial | hungarian visler | massaicattle | pumimudi | shorthairedsegugio | yorksire |
| caodecastrolaboreiro | englishspringerspaniel | hungarian vizla | masstif | pup | shorthr | yorky |
| cardigan corgi | englishstaffordshirebull | hungarian vizsla | masstiff | pyranean sheep | showzer | yorkypoo |
| cardigan welsh | enrichment | hungarian water | mastador | pyraneansheep | shpherd | yorshire |
| cardigancorgi | entelbuch mountain | hungarian wire hair | mastif | pyrean sheep | shranian | yoshon |
| cardiganwelsh | entelbuchmountain | hungarian wirehaird | mastiff | pyreansheep | shug | yourkie |
| carovcharka | entlebuch cattle | hungarian wirehaired | mastiif | pyrenan sheep | shui tzu | yrkie |
| carpathian | entlebuchcattle | hungarianhound | meliteo kinidio | pyrenansheep | shuitsu | yrkshire |
| carpatin | entlebucher | hungariankomondor | meliteokinidio | pyrenean mountain | shuitzu | yrksire |
| castro laboreiro | entlebucher mountain | hungariankuvasz | merle | pyrenean mountn | shutzu | yrt |
| castrolaboreiro | entlebucher sennenhund | hungarianmudi | mexican chinese crested | pyrenean mt | siberpoo | yugoslav mountain |
| catahoula | entlebuchermountain | hungarianpuli | mexican hairless | pyrenean sheep | sicilian | yugoslav shepherd |
| catalan sheep | entlebuchersennenhund | hungarianpulis | mexicanchinesecrested | pyrenean sheep long haired | sight hound | yugoslavian hound |
| catalansheep | epagneul breton | hungarianpumi | mexicanhairless | pyrenean sheep smooth faced | sighthound | yugoslavian shepherd |
| cattalan sheep | epagneulbreton | hungariansheep | minauture pinscher | pyrenean shepherd | sih doll | yugoslavianhound |
| cattalansheep | ernglish bull | hungarianshorthaired | minauturepinscher | pyreneanmountain | sih poo | yugoslavianshepherd |
| cattle australian | ernglishbull | hungarianvisla | mioretic sheep | pyreneanmountn | sih tzu | yugoslavmountain |
| cattle flanders bouvier | eskimo hound | hungarianvisler | mioreticsheep | pyreneanmt | sihdoll | yugoslavshepherd |
| cattle great swiss | eskimohound | hungarianvizla | mioritic sheep | pyreneansheep | sihpoo | zuchon |

***Table 3:*** *Summary statistics regarding tweets per phase, including length of phase (days); % representation of total tweets; total no. of tweets (organic and replies); total no. of twitter accounts contributing, mean no. of twitter accounts contributing (standard error and range) and mean no. of ‘favourites’ and ‘retweets’ per tweet.*

|  | **LOCKDOWN** | **PHASE EASE 1** | **PHASE EASE 2** | **PHASE EASE 3** |
| --- | --- | --- | --- | --- |
| Length of Phase (Days) | 51 | 19 | 33 | 50 |
| % of Total Tweets | 55.7 | 15.9 | 14.2 % | 14.2 % |
| Total No. of Tweets  (Organic, Replies) | 34028  (26689, 7339) | 9732  (7301, 2431) | 8671  (6165, 2506) | 8657  (5698, 2959) |
| Total No. of Twitter Accounts | 25462 | 8137 | 7088 | 7125 |
| Mean No. of Tweets per Day  (SE, Range) | 667.2  (20.9, 430-1036) | 512.2  (28.4, 343-956) | 262.8  (7.5, 179-377) | 173.1  (6.3, 101-313) |
| Mean No. of ‘Favourites’ and ‘Retweets’ per Tweet | 15.1, 3.2 | 18.1, 3.2 | 10.1, 2.2 | 16.0, 3.7 |

***Table 4:*** *Summary statistics regarding tweets per phase, per sector, including total no. of tweets (organic and replies); total no. of twitter accounts contributing, mean no. of tweets per day (standard error and range).*

|  | **LOCKDOWN** | **PHASE EASE 1** | **PHASE EASE 2** | **PHASE EASE 3** | **TOTAL** |
| --- | --- | --- | --- | --- | --- |
| **OTHER** | | | | | |
| Total No. of Tweets  (Organic, Replies) | 1753  (1651,102) | 487  (452, 35) | 523  (480, 43) | 397  (367, 30) | 3160 |
| Total No. of Twitter Accounts | 208 | 156 | 138 | 122 |  |
| Mean No. of Tweets per Day  (SE, Range) | 37.3  (2.3, 11-77) | 25.6  (1.6, 12-38) | 15.8  (1.5, 4-35) | 7.9  (0.5, 1-16) |  |
| **PERSONAL** | | | | | |
| Total No. of Tweets  (Organic, Replies) | 2543  (2421, 122) | 741  (697, 44) | 490  (454, 36) | 476 (439, 37) | 4250 |
| Total No. of Twitter Accounts | 365 | 256 | 193 | 178 |  |
| Mean No. of Tweets per Day  (SE, Range) | 54.1  (3.0, 16-113) | 39.0  (3.3, 19-78) | 14.8  (0.9, 7-31) | 9.5  (0.5, 3-20) |  |
| **PRESS** | | | | |  |
| Total No. of Tweets  (Organic, Replies) | 605  (599, 6) | 188  (188, 0) | 333  (329, 4) | 241  (239, 2) | 1367 |
| Total No. of Twitter Accounts | 97 | 62 | 72 | 66 |  |
| Mean No. of Tweets per Day  (SE, Range) | 12.9  (0.9, 5-28) | 9.9  (1.4, 3-30) | 10.7  (1.4, 1-35) | 4.9  (0.4, 1-15) |  |
| **STATE** | | | | | |
| Total No. of Tweets  (Organic, Replies) | 234  (225, 9) | 99  (97, 2) | 92  (91, 1) | 57  (57, 0) | 482 |
| Total No. of Twitter Accounts | 27 | 23 | 24 | 9 |  |
| Mean No. of Tweets per Day  (SE, Range) | 5.3  (0.4, 1-11) | 5.2  (0.5, 2-9) | 3.0  (0.3, 1-7) | 2.0  (0.2, 1-5) |  |
