# Supplementary material for: Pooches on a platform: Text mining twitter for sector perceptions of dogs during a global pandemic
Source: Front Vet Sci. 2023 Mar 1;10:1074542. doi: 10.3389/fvets.2023.1074542 (PMC10014727; doi:10.3389/fvets.2023.1074542)

***Figure 1:*** *Geocode buffer (Lat = 55.5166, Long = -4.0661, Buffer = 390 miles), allowing us to gather tweets from the United Kingdom (UK) and the Republic of Ireland (ROI) only (created within QGIS^97^).*

***
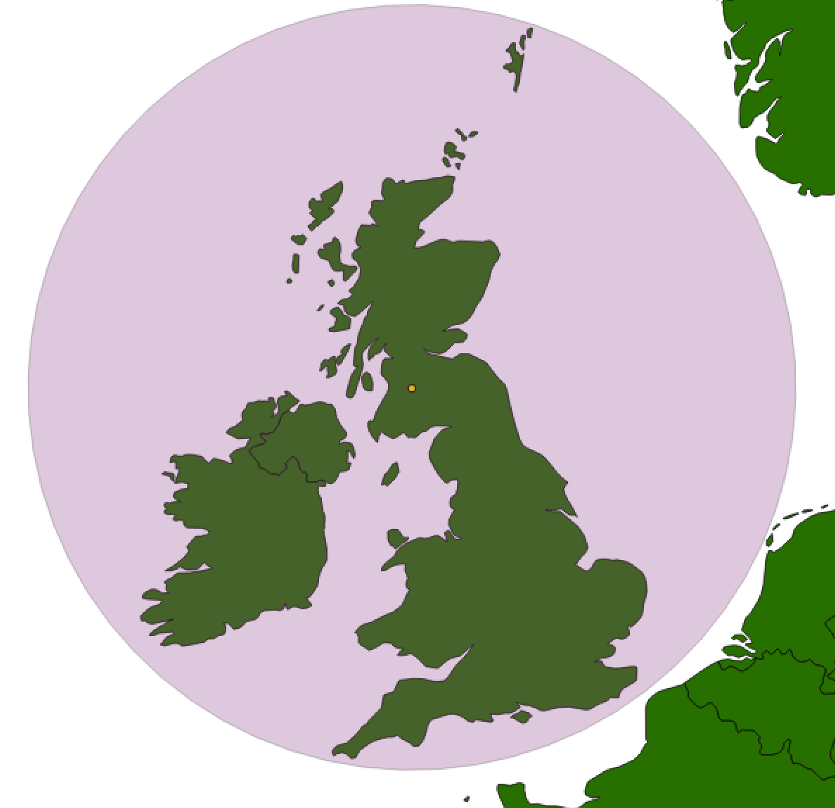
***

***Figure 2:*** *No. of original (black line) and reply (pink line) tweets published per day, across the full period (27^th^ March – 22^nd^ August 2020). The four phases are highlighted in block colours: Lockdown (27^th^ March - 12^th^ May 2020); Phase Ease 1 (13^th^ May – 31^st^ May 2020); Phase Ease 2 (1^st^ June – 3^rd^ July 2020), and Phase Ease 3 (4^th^ July - 22^nd^ August 2020). Related summary statistics are listed in Supplementary Table 3.*


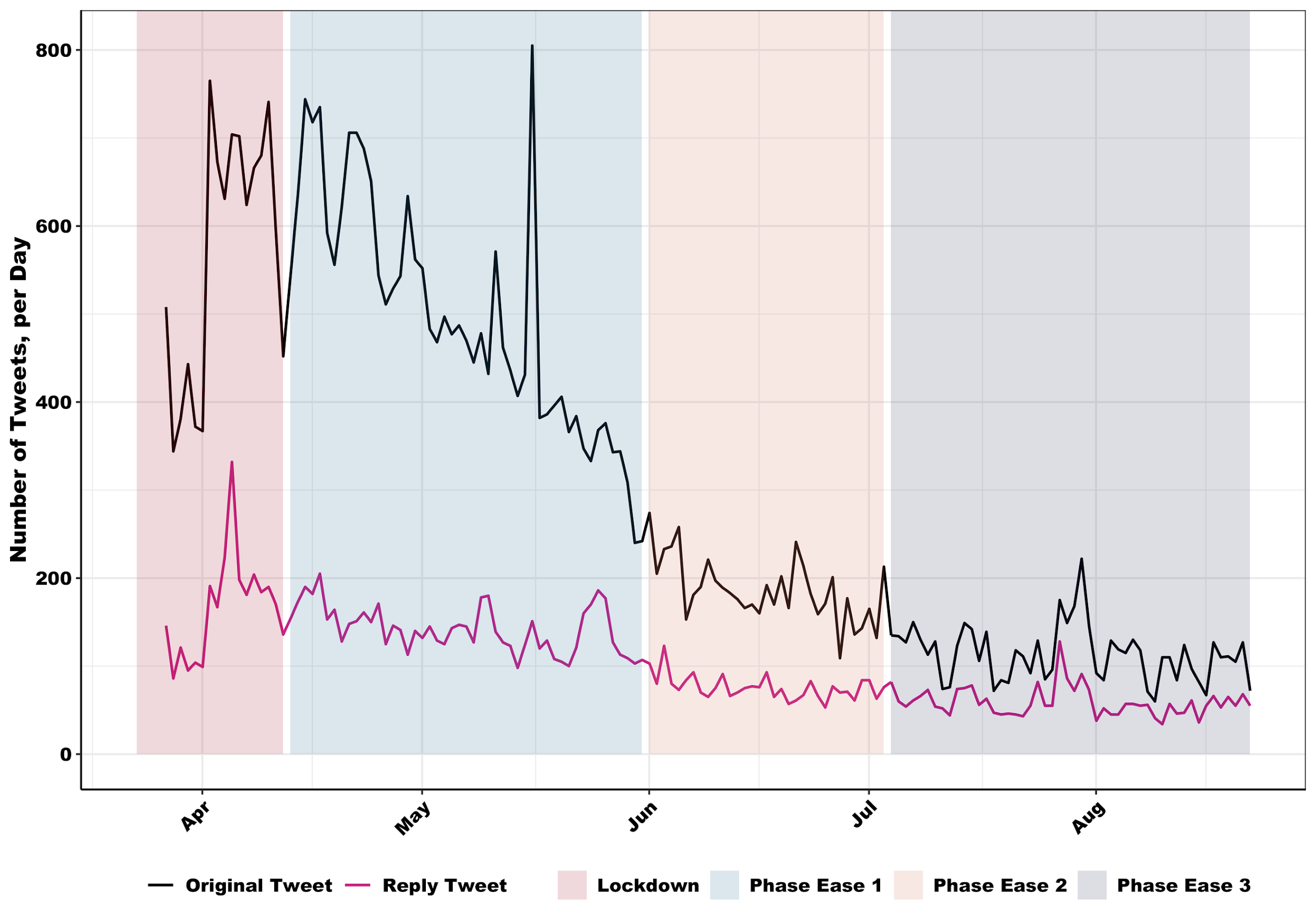


***Figure 3:*** *Top 50 frequently used hashtags, in original tweets, across the full period (27^th^ March – 22^nd^ August 2020).*


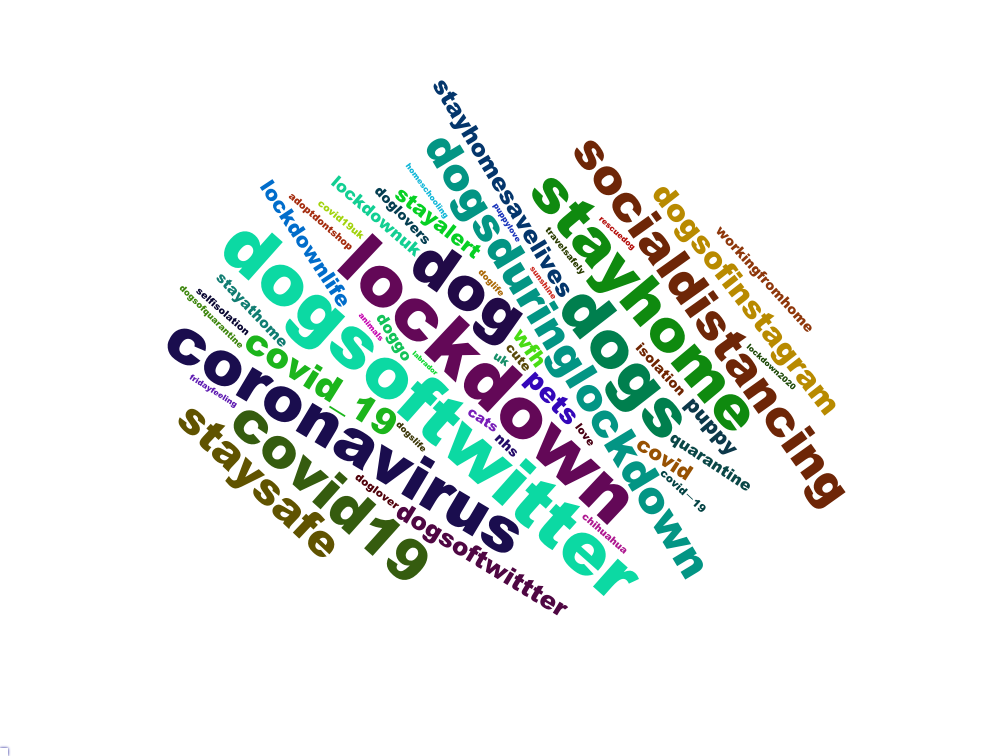


***Figure 4:*** *Most frequently used single words (or tokens), per phase: Lockdown (27^th^ March - 12^th^ May 2020); Phase Ease 1 (13^th^ May – 31^st^ May 2020); Phase Ease 2 (1^st^ June – 3^rd^ July 2020), and Phase Ease 3 (4^th^ July - 22^nd^ August 2020). ‘Walk’ remained the most frequently used token, while ‘distance’, ‘home’, ‘like’, ‘love’, ‘work’, ‘help and ‘need’ remained consistently well used (among others). Word stemming has been applied.*


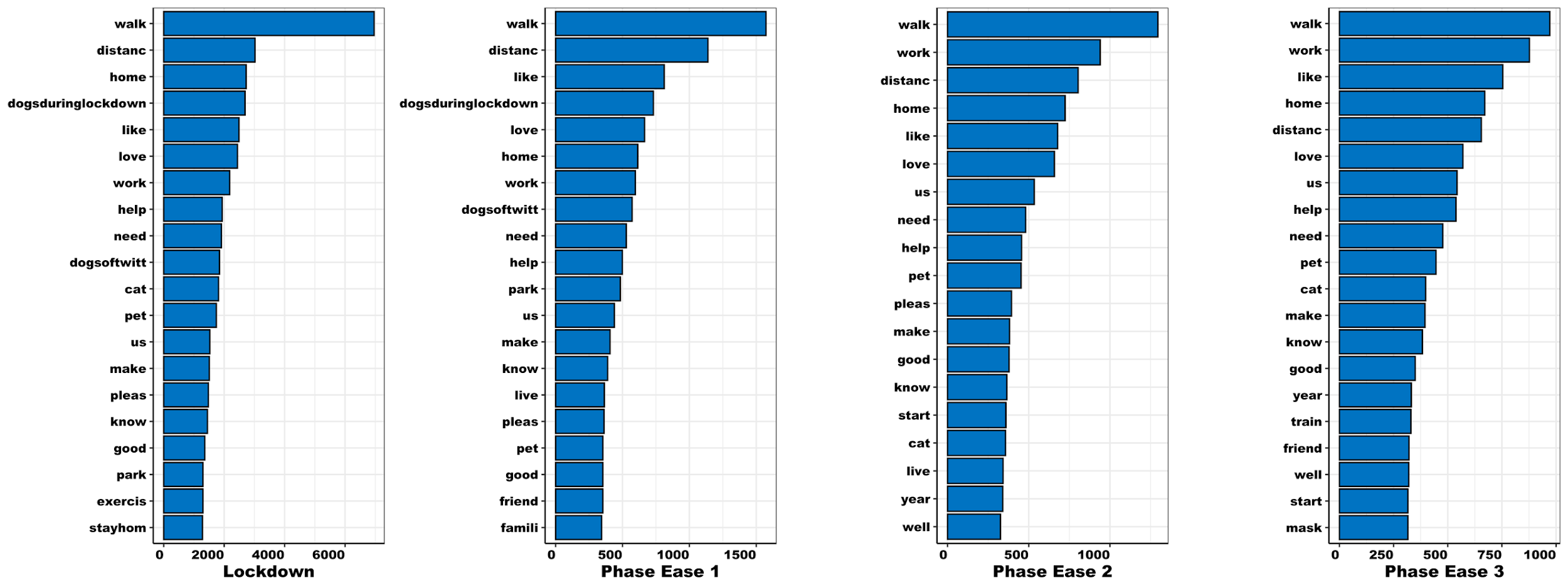


***Figure 5:*** *Top fifteen sentiment-associated words (afinn lexicon), across the full period (27^th^ March – 22^nd^ August 2020), that had the greatest contribution to sentiment values, in either a positive (blue) or negative (red) direction. This is computed by multiplying their sentiment value by the number of times they appear. It is important to note that frequently used tokens ‘help’, ‘like’ and ‘love’ (highlighted in Supplementary Figure 4) are present, thus their contribution to overall sentiment should be considered with caution.*


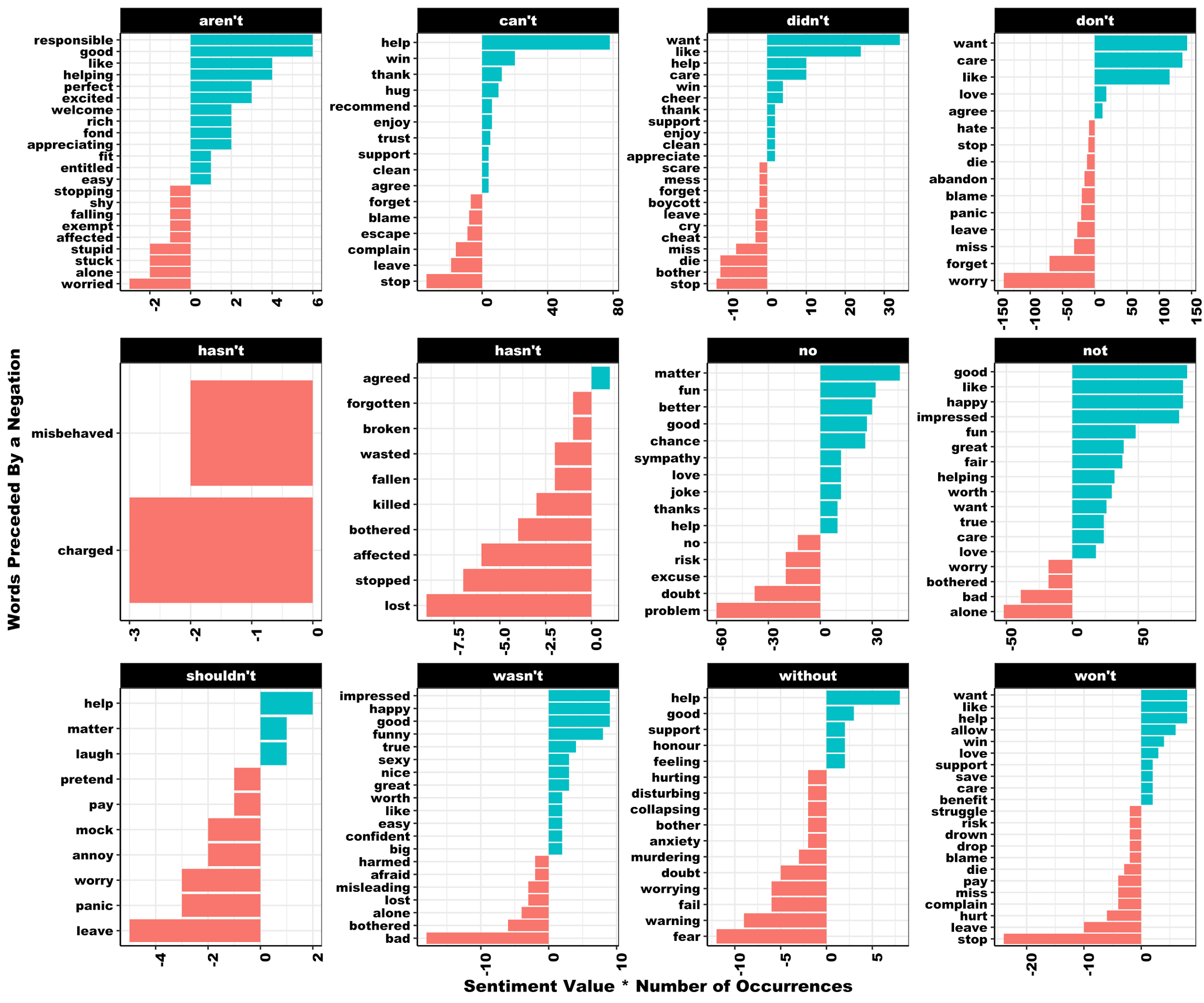


***Figure 6:*** *Most frequently used bigrams, per phase: Lockdown (27^th^ March - 12^th^ May 2020); Phase Ease 1 (13^th^ May – 31^st^ May 2020); Phase Ease 2 (1^st^ June – 3^rd^ July 2020), and Phase Ease 3 (4^th^ July - 22^nd^ August 2020). Prominent topics include (but are not limited to): rules/safety (e.g., ‘stay safe’ and ‘stay alert’); exercise/walking (e.g., ‘daily exercise’ and ‘daily walk’); meat trade (e.g., ‘cat meat’ and ‘meat trade’); canine focused (e.g., ‘separation anxiety’ and ‘greyhound/racing stay/safe/race/safe’); practicalities of lockdown (e.g., ‘ticket online’ and ‘contactless payment’); location specific topics (e.g., ‘chinese city’ and ‘south korean’); travel/public transport (e.g., ‘public transport’ and ‘more/travel/safely stay/alert’); medical advances (‘uk researchers’, ‘detect signs’ and ‘test positive’); mental health (e.g., ‘mental health’); employment (e.g., ‘unemployment payment’, ‘staff sign’ and ‘nhs staff’); outdoor space (e.g., ‘fresh air’) and non-specific topics (e.g., ‘moon live’ and ‘world sign’). Bigrams that appear in all panels include meat trade (including meat festival) and separation anxiety.*


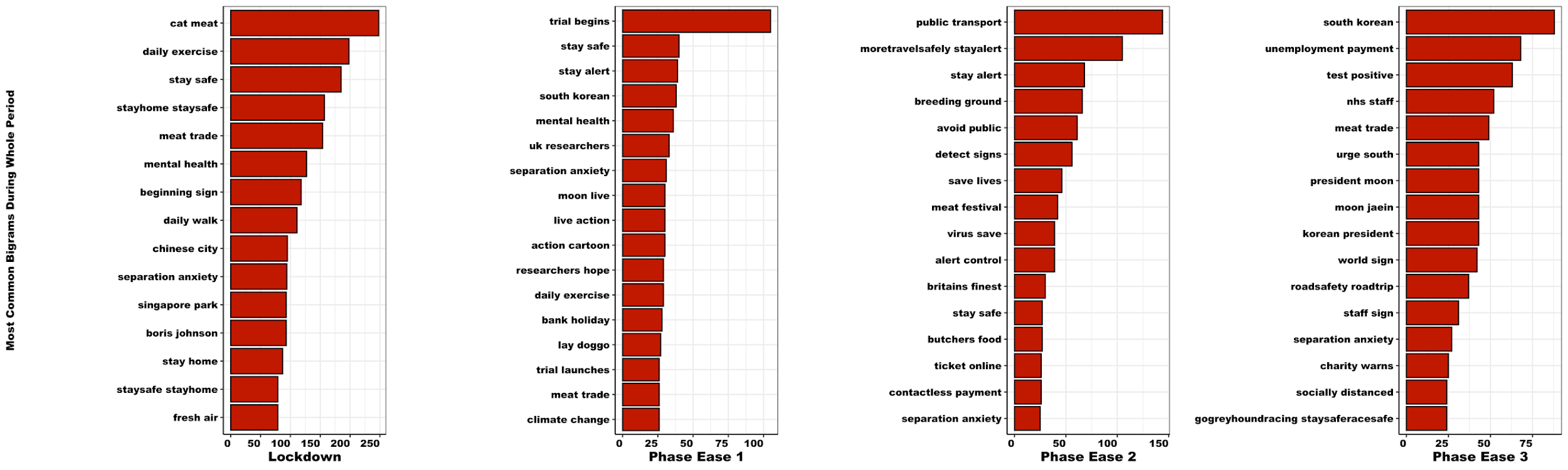


***Figure 7:*** *‘nrc’ sentiment scores, per phase: Lockdown (27^th^ March - 12^th^ May 2020); Phase Ease 1 (13^th^ May – 31^st^ May 2020); Phase Ease 2 (1^st^ June – 3^rd^ July 2020), and Phase Ease 3 (4^th^ July - 22^nd^ August 2020). Note the lower overall sentiment during Phase Ease 1 and Phase Ease 3. Phase Ease 3 exhibits an increase in* *‘anger’, ‘sadness’, ‘fear’ and ‘negative’ sentiment, while also showing a decrease in ‘joy’ and ‘positivity’.*


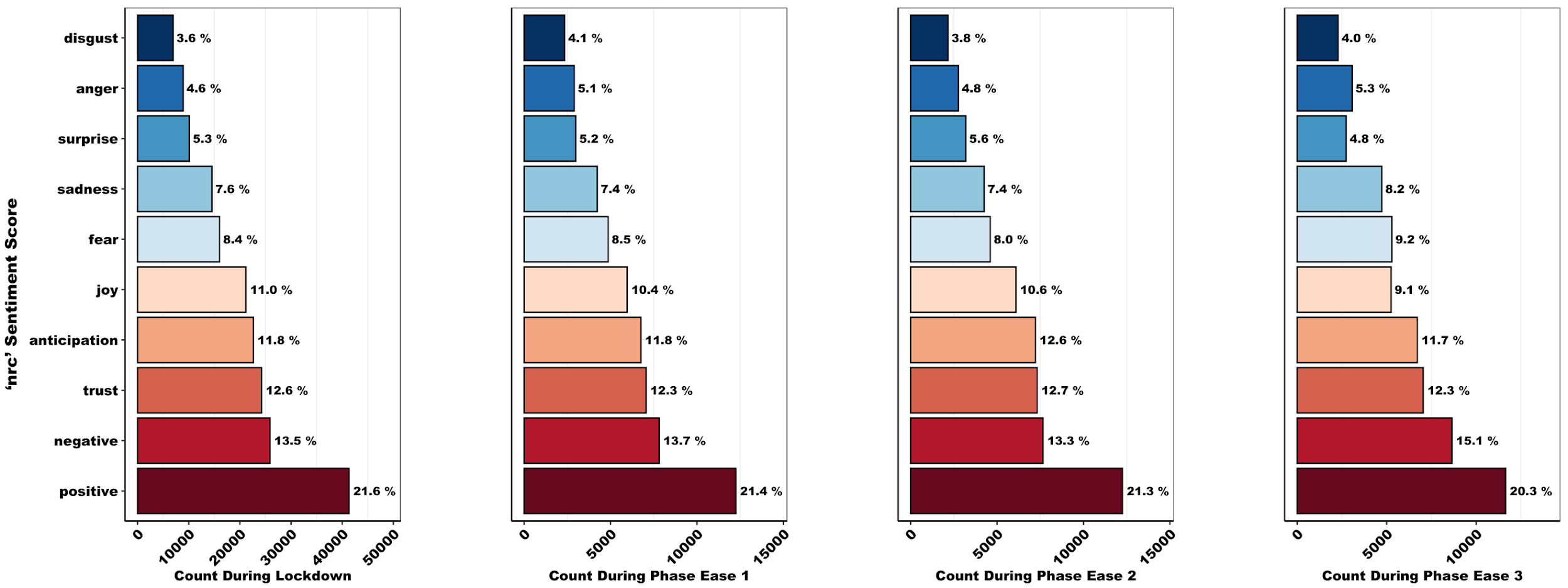


***Figure 8:*** *‘bing’ sentiment scores, per phase: Lockdown (27^th^ March - 12^th^ May 2020); Phase Ease 1 (13^th^ May – 31^st^ May 2020); Phase Ease 2 (1^st^ June – 3^rd^ July 2020), and Phase Ease 3 (4^th^ July - 22^nd^ August 2020). Note the lower total cumulative and mean sentiment score during Phase Ease 3.*


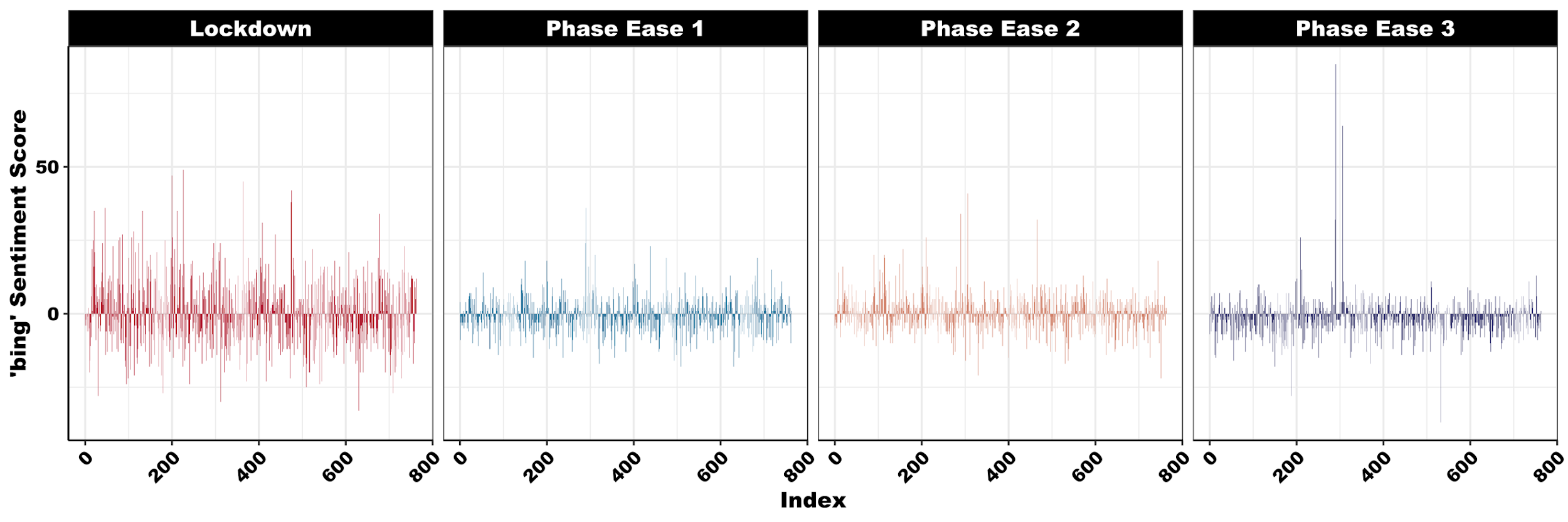


|  | **‘*bing*’ Sentiment** | | | | | **Positive** | | **Negative** | |
| --- | --- | --- | --- | --- | --- | --- | --- | --- | --- |
|  | *Total (cumulative)* | *Mean (SE)* | *Median* | *Maximum* | *Minimum* | *Maximum* | *Minimum* | *Maximum* | *Minimum* |
| **Lockdown** | 118 | 0.15 (0.42) | -1 | 49 | -33 | 72 | 6 | 74 | 3 |
| **Phase Ease 1** | -476 | -0.62 (0.22) | -1 | 36 | -18 | 46 | 0 | 29 | 0 |
| **Phase Ease 2** | 117 | 0.15 (0.22) | 0 | 41 | -22 | 46 | 0 | 34 | 0 |
| **Phase Ease 3** | -1233 | -1.62 (0.24) | -2 | 85 | -37 | 100 | 0 | 49 | 0 |

***Figure 9:*** *Comparing word frequencies of Phase Ease 3 with all other phases: Lockdown (27^th^ March - 12^th^ May 2020); Phase Ease 1 (13^th^ May – 31^st^ May 2020); Phase Ease 2 (1^st^ June – 3^rd^ July 2020), and Phase Ease 3 (4^th^ July - 22^nd^ August 2020). Pearson correlation coefficient noted in top left of panel). Words that are far from the line identify those that are found more in one set of texts than another. Words extend to lower frequencies for PE3-LD comparison, which indicates that PE3 and LD use more similar wording than PE3-PE1 or PE3-PE2.*


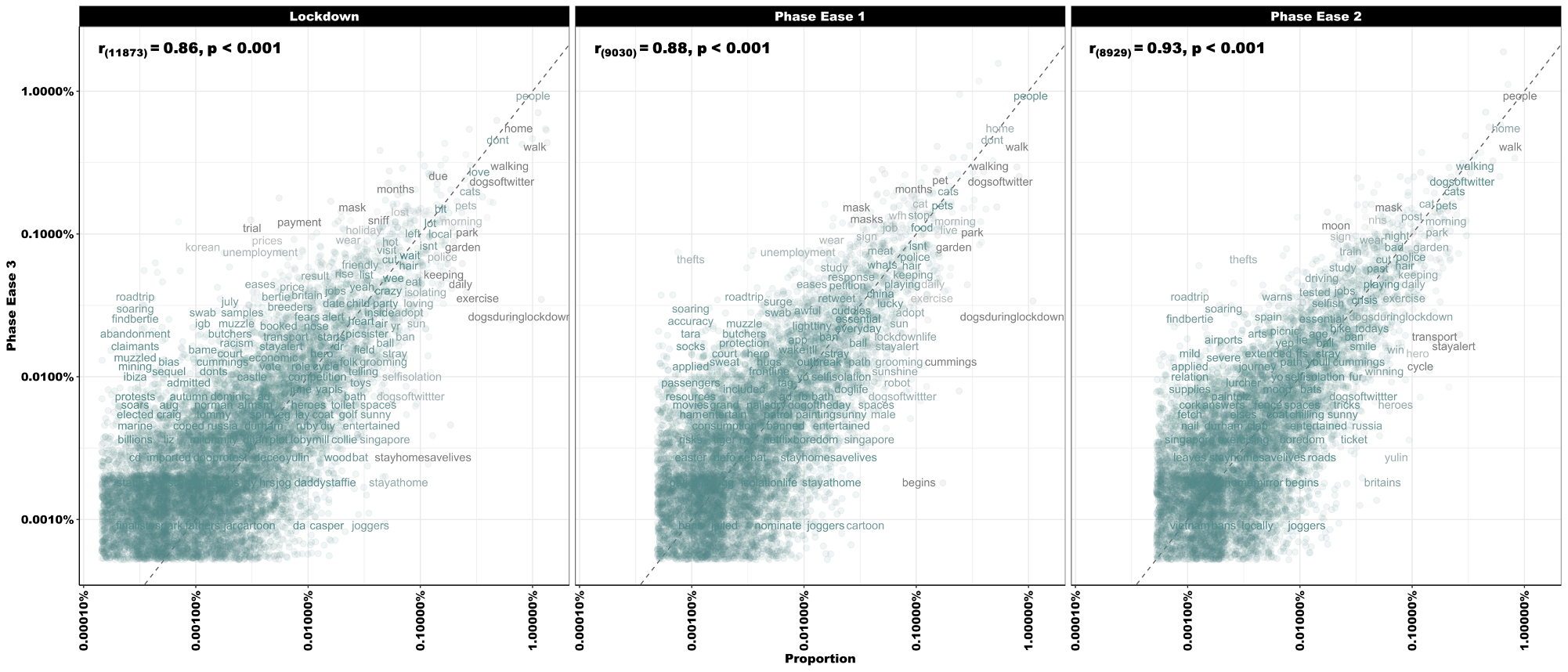


# ***Figure 10:*** *Results from TF-IDF analyses, identifying words that are important to one phase within a collection of the full period. PE3 focuses more heavily on rules/safety issues including: ‘keep/your/dog/safe’, ‘road/safety’ and ‘thefts’. Whereas topics present for LD include ‘dogs/of/quarantine’ and ‘national/pet/day’ (occurring during the phase). PE1 focused on ‘mental/health/awareness/week’, ‘more/travel/safely’ (travel/public transport) and ‘last/normal/photo’ (sharing last image before lockdown). PE1 also introduces the importance of outdoors space (‘foot/paths/more’) and communities (‘communities/they’). The top occurrences for PE2 include ‘bring/your/dog/to/work/day’ and an increased interest in cycling (‘cycle/find’) and ‘volunteers/week’. Additionally, more emotive topics become evident during PE2, e.g., ‘this/calls/for/love’ and ‘help/in/it/together’.*


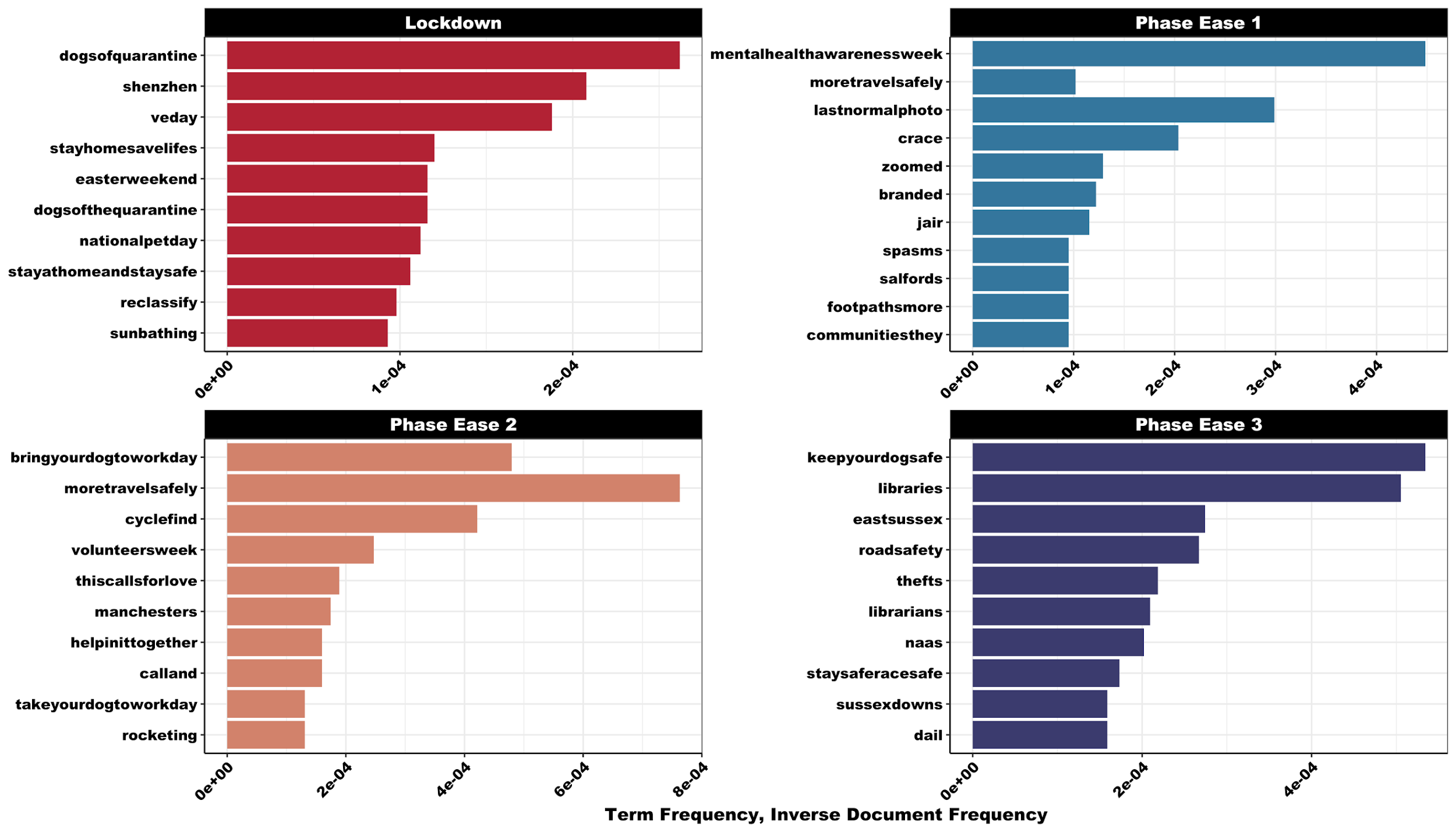


*
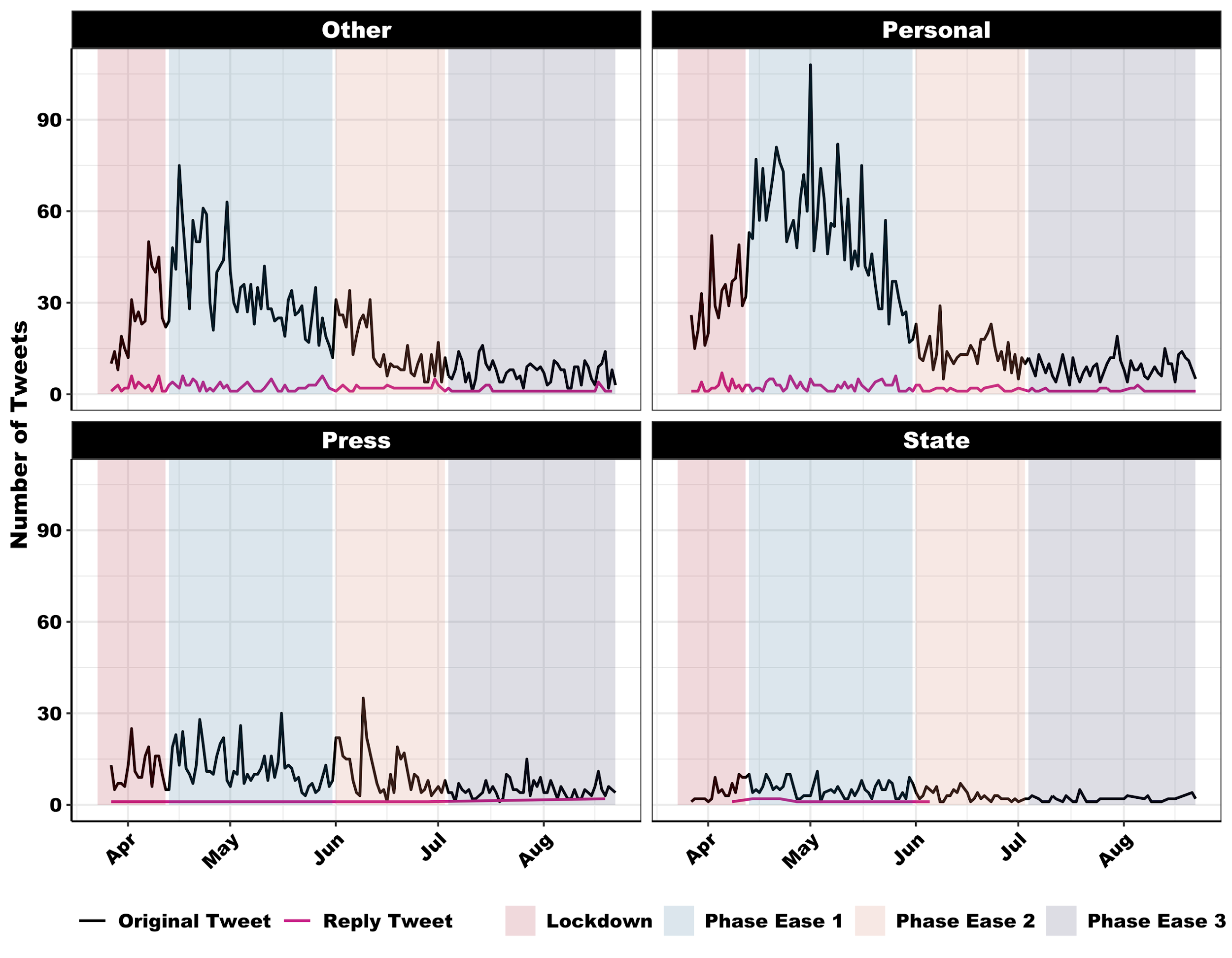
****Figure 11:*** *Number of original (black line) and reply (pink line) tweets published per day, across the full period, for each sector: (1) “Other” i.e., all other sectors; (2) “Personal” i.e., personal accounts; (3) “Press” i.e., all variations of mass media (online and offline); (4) “State” i.e., Government, Police and NHS. The four phases are highlighted in block colours: Lockdown (27^th^ March - 12^th^ May 2020); Phase Ease 1 (13^th^ May – 31^st^ May 2020); Phase Ease 2 (1^st^ June – 3^rd^ July 2020), and Phase Ease 3 (4^th^ July - 22^nd^ August 2020). Related summary statistics are listed in Supplementary Table 4.*

*
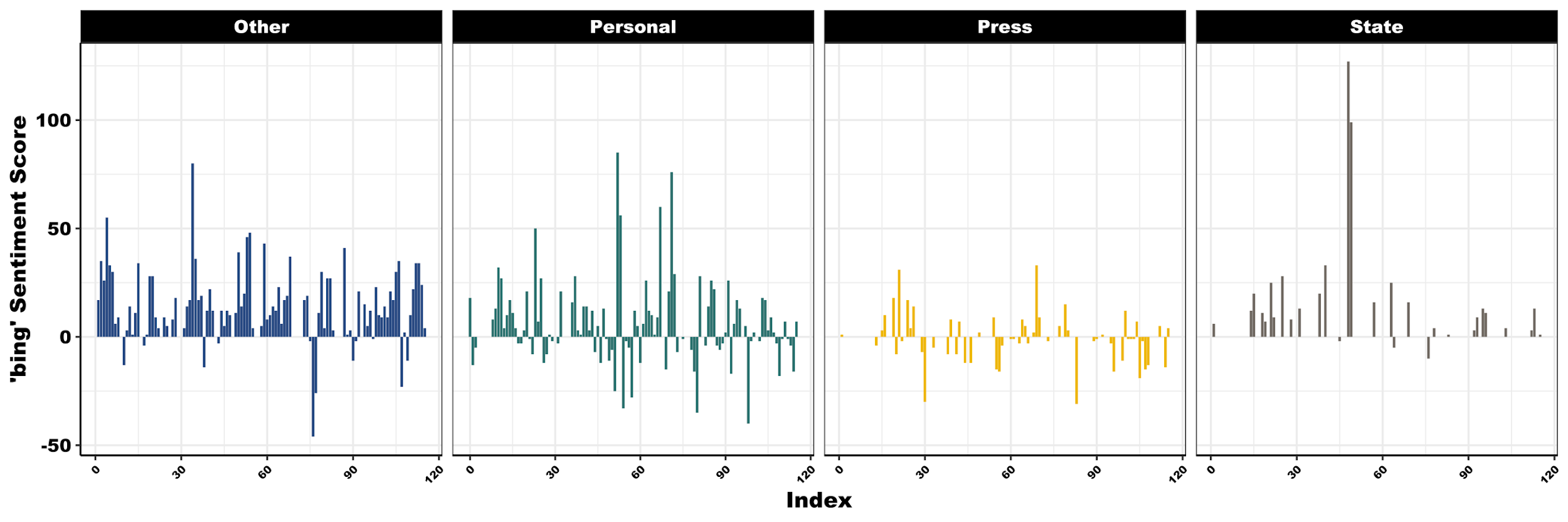
****Figure 12:*** *‘bing’ sentiment scores, across the full period (27^th^ March – 22^nd^ August 2020), per sector: 1) “Other” i.e., all other sectors; (2) “Personal” i.e., personal accounts; (3) “Press” i.e., all variations of mass media (online and offline); (4) “State” i.e., Government, Police and NHS. Note the lower total cumulative and mean sentiment score for Press accounts.*

|  | **‘*bing*’ Sentiment** | | | | | **Positive** | | **Negative** | |
| --- | --- | --- | --- | --- | --- | --- | --- | --- | --- |
|  | *Total (cumulative)* | *Mean (SE)* | *Median* | *Maximum* | *Minimum* | *Maximum* | *Minimum* | *Maximum* | *Minimum* |
| **Other** | 1410 | 14.39 () | 12 | 80 | -46 | 108 | 0 | 89 | 0 |
| **Personal** | 614 | 5.90 () | 3 | 85 | -40 | 94 | 2 | 66 | 0 |
| **Press** | -38 | -0.64 () | -1 | 33 | -31 | 42 | 0 | 58 | 0 |
| **State** | 520 | 16.25 () | 10 | 127 | -10 | 173 | 0 | 46 | 0 |

***Figure 13:*** *Most frequently used single words (or tokens), across the full period (27^th^ March – 22^nd^ August 2020), per sector: (1) “Other” i.e., all other sectors; (2) “Personal” i.e., personal accounts; (3) “Press” i.e., all variations of mass media (online and offline); (4) “State” i.e., Government, Police and NHS. ‘Walk’ and ‘help’ remain consistently well used. However, popular hashtags, e.g., ‘dogs/during/lockdown’ and ‘dogs/of/twitter’, and more emotive language e.g., ‘love’ and ‘like’ are only present within Other and Personal accounts. Press accounts frequently use wording regarding ‘pet’ and ‘owner’ e.g., ‘train’, ‘warn’, ‘food’, ‘tip’. Meanwhile State accounts refer to restrictions and public spaces often e.g., ‘park’, ‘distanc-’, ‘bin’, ‘rule’ etc. Word stemming has been applied.*


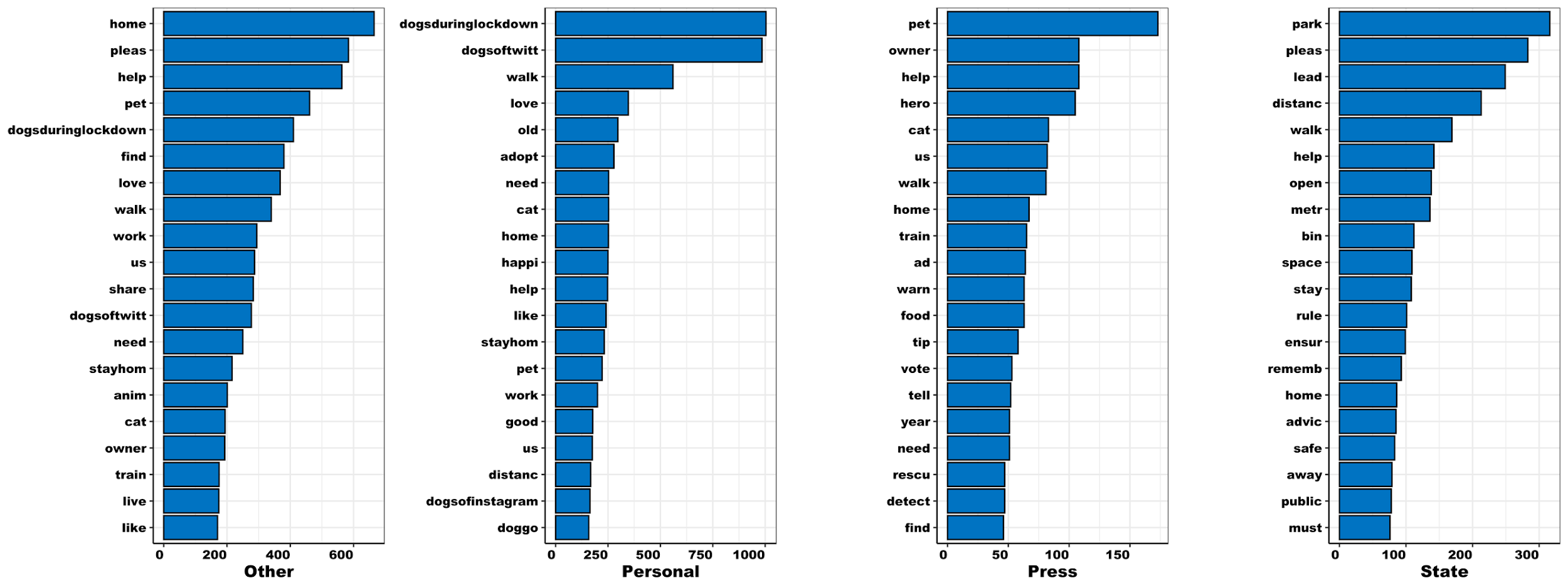

Supplement: Supplementary file 2 [file Data_Sheet_2.docx]
