# Supplementary material for: Pooches on a platform: Text mining twitter for sector perceptions of dogs during a global pandemic
Source: Front Vet Sci. 2023 Mar 1;10:1074542. doi: 10.3389/fvets.2023.1074542 (PMC10014727; doi:10.3389/fvets.2023.1074542)
Supplement: Supplementary file 3 [file Data_Sheet_3.docx]

***Note 1:*** *Limitations of the study.*

Whilst there is great potential from analyses of text-mined twitter data, this work is not without its limitations. With regards to sampling, tweets were searched using two term lists: (1) words related to the COVID-19 and (2) words related to dogs, and relevance was checked by searching for the presence of one or more pre-defined terms. Twitter users participating in canine-COVID conversations may not have adhered these search assumptions – thus, will have been excluded from the analyses^1^. We suggest that future research apply Natural Language Processing (NLP) tools, e.g., Bidirectional Encoder Representations from Transformers (BERT) language models which have achieved outstanding results regarding text recognition and classification^2,3^.

A second sampling issue is associated with the common use of Twitter’s free Streaming API for sampling tweets. Research into the Twitter Streaming API has highlighted troubling implications for the standard approaches when gathering Twitter data using search word(s). For example, when the free streaming API was compared with paid access to the Firehose API, the sample became less representative as the number of parameters requested increased^4-6^. This emphasises two issues: samples of selected keywords are not representative of the data, and methodologies surrounding sampling are not transparent. However, in January 2021, Twitter announced the “Academic Research Product Track”, which provides access to the Twitter v2 API endpoints (introduced in 2020), as well as much improved data access. In summary the product track allows the authorized user: (1) access to the full archive of tweets published on Twitter; (2) a higher monthly tweet cap (20x what was previously possible with the standard v1.1 API); and (3) ability to access these data with more precise filters permitted by the v2 API^7^. Consequently, this new track may limit the troubling implications mentioned above.

Finally, for the purposes of this study, the delineation of the population into one of four sectors was carried out by manually accessing the Twitter bio of all accounts where 4 or more relevant tweets were posted during the full period (27th March - 22nd August 2020). The Twitter bio is a small public summary regarding account holder or business. First, this biases the data towards users participating in debates/conversations regularly. Secondly, if the bio does not accurately represent the account, sector categorisation will be inappropriate. And third, while the categorisation is broad enough to encompass most sectors, finer scale variation will be lost. Finally, Twitter is not only the social media of choice for Other, Press, Public or State users, thus it is not assumed to be representative of whole population.

**REFERENCES for *Note S1***

1. Marres N, Moats D. Mapping controversies with social media: The case for symmetry. Soc Media Soc. (2015) 1(2): 2056305115604176. doi: 10.1177/2056305115604176
2. Klein AZ, Magge A, O’Connor K, Amaro JIF, Weissenbacher D, Henandez GG. Toward using twitter for tracking covid-19: A natural language processing pipeline and exploratory data set. J Med Internet Res. (2021) 23(1): e25314. doi: 10.2196/preprints.25314
3. Pota M, Ventura M, Catelli R, Esposito M. An effective BERT-based pipeline for Twitter sentiment analysis: a case study in Italian. Sensors (2021) 21(1): 133. doi: 10.3390/s21010133
4. Campan A, Atnafu T, Truta TM, Nolan J. Is data collection through twitter streaming api useful for academic research? 2018 IEEE International Conference on Big Data (Big Data) (2018). IEEE.
5. Bruns A, Liang YE. Tools and methods for capturing Twitter data during natural disasters. First Monday (2012).
6. van Vliet L, Törnberg P, Uitermark J. The Twitter parliamentarian database: Analyzing Twitter politics across 26 countries. PloS One (2020) 15(9): e0237073. doi: 10.1371/journal.pone.0237073
7. Barrie C, Ho JCT. academictwitteR: an R package to access the Twitter Academic Research Product Track v2 API endpoint. J Open Source Softw. (2021) 6(62): 3272. doi: 10.21105/joss.03272
